# Collective Locomotion of Human Cells, Wound Healing and Their Control by Extracts and Isolated Compounds from Marine Invertebrates

**DOI:** 10.3390/molecules25112471

**Published:** 2020-05-26

**Authors:** Claudio Luparello, Manuela Mauro, Valentina Lazzara, Mirella Vazzana

**Affiliations:** Department of Biological, Chemical and Pharmaceutical Sciences and Technologies (STEBICEF), University of Palermo, 90128 Palermo, Italy; manuela.mauro01@unipa.it (M.M.); valentina.lazzara@community.unipa.it (V.L.); mirella.vazzana@unipa.it (M.V.)

**Keywords:** marine invertebrates, cell migration, wound healing

## Abstract

The collective migration of cells is a complex integrated process that represents a common theme joining morphogenesis, tissue regeneration, and tumor biology. It is known that a remarkable amount of secondary metabolites produced by aquatic invertebrates displays active pharmacological properties against a variety of diseases. The aim of this review is to pick up selected studies that report the extraction and identification of crude extracts or isolated compounds that exert a modulatory effect on collective cell locomotion and/or skin tissue reconstitution and recapitulate the molecular, biochemical, and/or physiological aspects, where available, which are associated to the substances under examination, grouping the producing species according to their taxonomic hierarchy. Taken all of the collected data into account, marine invertebrates emerge as a still poorly-exploited valuable resource of natural products that may significantly improve the process of skin regeneration and restrain tumor cell migration, as documented by in vitro and in vivo studies. Therefore, the identification of the most promising invertebrate-derived extracts/molecules for the utilization as new targets for biomedical translation merits further and more detailed investigations.

## 1. Introduction

The collective migration of cells is a complex integrated process at the basis of several different physiological events, such as embryo development, tissue shaping, and wound healing, whereas, in the course of diseases, like carcinogenesis, it is strongly implied in the step of tumor cell metastatization. In the recent years, a great deal of literature papers has focused on the molecular, biophysical and cytological aspects of cell locomotory patterns in the awareness of the clinical relevance shown by the advancement of knowledge on the mechanism and dynamism of collective cell migration. These studies shed light upon the mechanobiological bases of movement generation and speed/orientation setting and unveiled some of the signal transduction properties that are linked to the biochemical features of the migratory process. As a result of this research, the roles played in epithelial-mesenchymal transition (EMT) by integrin, junction protein and cytoskeletal dynamics, growth factor/cytokine and metalloprotease secretion, as well as cell interactions with components of the extracellular matrix (ECM) have been increasingly elucidated. A comprehensive overview of these topics can be found in several previously-published reviews [1,2,3].

Among the events of tissue repair involving collective cell motility, skin wound healing represents a multi-step coordinated process, which, following the initial inflammatory and hemostatic stages, associates re-epithelization, i.e., epidermal cell locomotion and growth on top of the granulation tissue, with tissue recovery implying the restoration of vascularization via angiogenesis and fibroplasia, which is the recruitment of activated fibroblasts and newly-differentiated myofibroblasts for ECM reconstitution and wound contraction [4,5,6,7,8]. Once the wound damage is repaired, a final tissue remodeling is undertaken to restore skin integrity and structure [9,10]. During wound re-epithelization, human keratinocytes move in a spatially- and directionally-organized way, whose molecular basis can be found in specific cell-ECM interactions, like the binding of type XVII collagen by both intermediate filament and actin cytoskeleton via hemidesmosomal actinin 4, and the creation of gradients of epidermal growth factor (EGF) immobilized within the ECM and presented to cell receptors [11,12]. Indeed, EGF is one of the most potent regulators of keratinocyte locomotory behavior along with transforming growth factor (TGF)-β, whose prominent regulatory effect on epidermal cell motility during wound repair via ERK signaling pathways is widely acknowledged [13]. Scientific interest has also focused upon the transcriptional factors that are up-regulated and activated in migrating keratinocytes during the repair process, leading to the identification, among others, of Smad7, Stat3, and c-Jun, which are functionally involved in the promotion of cutaneous wound healing, as extensively reviewed by Boudra and Ramsey [14].

**Figure 1:**
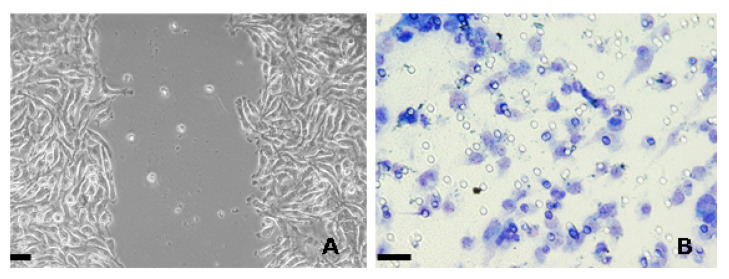
(**A**) Phase-contrast microscopic image of a scratch created in a monolayer of HEPG2 liver carcinoma cells. (**B**) Light microscopic image of MDA-MB231 breast carcinoma cells migrated through the pores of a polycarbonate filter in a Boyden chamber assay, fixed with 95% ethanol, and stained with 0.02 g toluidine blue/mL. Bar = 10 μm.

Collective cell motility can be studied both in culture and in vivo. In the first case, the migratory behaviour of cell monolayers can be evaluated via the “in vitro wound healing” or “scratch” assay, in which a gap is created by scratching a pipette tip through a confluent sheet of cells in culture (Figure 1A), and the eventually-occurring bidimensional cell migration leading to the closure of the scratch being recorded by either phase-contrast microscopic images captured at regular intervals or time-lapse videomicroscopy [15].

When the model system studied is a tumor cell line, the scratch assay is commonly complemented by either the “Boyden chamber” or the “transwell migration/invasion” assays, as originally developed by Albini and coworkers [16], which measure the ability of a population of neoplastic cells to migrate individually through a physical barrier, i.e., an untreated or basement membrane (Matrigel)-coated porous polyester membrane, in response to a chemoattractant or a motility-promoting compound (Figure 1B) [17,18].

Dealing with in vivo experimental systems, the group locomotion of tumor cells might be examined through the chick embryo chorioallantoic membrane (CAM) assay, which implies the grafting of neoplastic cells on the CAM, their subsequent interaction with the blood vessels, and the invasion of the adjacent mesenchymal tissue, which can be studied by microscopical, immunohistochemical, and molecular techniques [19]. CAM might be used also to study the angiogenetic process that is characterized by migration of mesodermal blood vessels into the basement membrane and the ECM leading to their compartmentalization into new vessels in the ectoderm [20]. The incision of the rodent dorsum has been commonly used as an in vivo model to study the effect of molecules and extracts on the whole process of skin repair. Using this technique, the evolution of wound closure can be macroscopically assessed at different time points and, once the animals are sacrificed, histological observations, immunohistochemical analyses, biophysical evaluations, such as the quantitation of tensile strength, and biochemical/molecular assays can be performed on the biopsies. This enables collecting additional multi-perspective information on the fine tuning of tissue repair and also to evaluating the ability of supplemented substance(s) to promote a quick, robust, and histologically-shaped healing aimed to obtain the most correct skin replacement [21]. In vivo burn injury models may be created by pressing for few seconds a shaved and stretched area of the rodent dorsum skin with a metal block pre-heated at 100 °C or more, thus enabling studying the effect of novel compounds for topical therapeutics that are designed for the treatment of burn lesions [22]. Progresses in the knowledge of the molecular bases of wound repair and in the design of novel more efficacious medicaments are of basic importance to develop effective treatments for compromised healing occurring in pathologies, such as diabetes, arteriosclerosis, malnourishment, and malignant tumors. However, it is worth-mentioning that, although small animal models are commonly used for wound healing research, they display some biological limitations due to the differences in the histological architecture of rodent vs. human skin, and in the primary physiological act of healing, i.e., myofibroblast contraction for rodents vs. re-epithelization for humans. Other experimental models, such as the porcine one, have been proven to be more linkable with the human healing process, but the costs of housing and the difficulties in handling have limited their usability for first-line exploratory research [23,24]. Another aspect to consider is that “pure” scarring-free regeneration, which only sees new cells completely replacing the lost ones, is very uncommon in humans. In fact, regeneration is a well-known phenomenon in which animals restore lost parts of their body through four overlapping steps: (a) wound healing and histolysis, (b) blastema formation, (c) recognition of positional information, and (d) repatterning of the missing portion. As discussed by Krafts [25], the repair process in the context of the healing of human tissues consists of both regeneration and replacement, with the latter seeing the intervention of inflammatory cells and the sequential formation of granulation and fibrous tissues, which make the biological system more multifaceted.

It is widely recognized that seas and oceans, which account for about 97% of the whole water in the world, have a still little exploited asset in terms of biodiversity and, consequently, marine species-derived biological products. Much research interest has been focused on marine invertebrates, which represent more than 50% of the species inhabiting the aquatic environment in Europe. A remarkable amount of secondary metabolites is produced by aquatic invertebrates to aid in both the inter-individual signalization and the defense from predators, competitors, infective agents and UV radiation damages due to the adaptation mechanisms to the specific life conditions in the considerably differing marine ecosystems. An ever increasing number of these substances displays active pharmacological properties with an extensive spectrum of indications, making them prospective drugs of marine origin to be developed for therapeutic applications [26]. Within this context, the aim of this review is to pick up selected studies that report the extraction and identification of mixed fractions or isolated chemicals which exert a modulatory effect on collective cell migration and/or skin tissue reconstitution and recapitulate the molecular, biochemical, and/or physiological aspects, where available, which are associated to the metabolites or extracts under examination. Such compounds will be listed respecting the taxonomic hierarchy of the producing invertebrate species in dedicated paragraphs, in which the structural aspects of each organism and their contribution to “blue biotech” applications, if any, will also be briefly outlined.

## 2. Porifera

Porifera is the oldest metazoan group that is still widespread on the planet. They are very simple animals, which are devoid of real tissues or organs, and only consist of a few types of specialized cells that are surrounded by a collagen-rich extracellular matrix [27].

**Figure 2:**
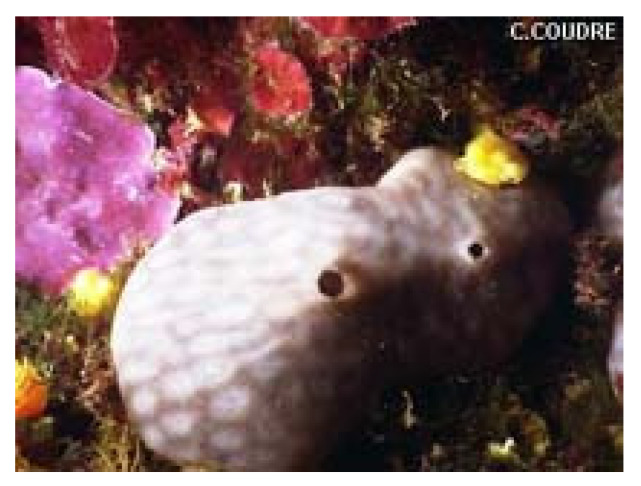
A specimen of *Chondrosia reniformis* demosponge. © Bernard Picton (CC BY-NC-SA 4.0); http://www.marinespecies.org/.

*Chondrosia reniformis* (Nardo, 1847; Demospongiae, Chondrosiida: Chondrosiidae) is a sessile species that lives in the shallow coasts of the Mediterranean Sea and the South-West coast of the Atlantic Ocean, which can reach a considerable body thickness (Figure 2).

This species shows remarkable regenerative properties and, for this reason, has been chosen as a model system for studies on the molecular pathways of tissue restoration in lower metazoan, highlighting the involvement of TGF superfamily proteins [28]. Unlike other sponges, this invertebrate lacks the silica spicules that commonly strengthen the other species’ body, instead obtaining its stiffness through a greater amount of collagen fibers that are tightly packed in a complex fibrillar network. Collagen extracts from *C. reniformis* have shown considerable potentiality in carrying out the role of nanoparticle vector and drug-coating material [29,30] in the design of biocompatible scaffolds for regenerative medicine [31]. Moreover, sticky hydrogels with self-healing properties have been obtained from collagen extracted from the ectosome and choanosome of this demosponge showing remarkable rheological properties that make them suitable as injectable matrices for biomedical applications [32]. Particular interest has been given to the cosmetic and wound healing effect of trypsin-digested *C. reniformis* collagen hydrolysates. In their paper, Pozzolini et al. [33] isolated different peptide fractions by HPLC and demonstrated that those ones particularly enriched in hydroxyproline-containing collagenous peptides promoted the migration of HaCaT keratinocytes and L929 fibroblasts in “scratch” assays, also accelerating cell proliferation at the site of the wound with respect to the untreated controls at the same time points. In addition, these preparations appeared able to trigger type I collagen synthesis and extracellular release by fibroblasts and protect keratinocytes from UV damage by enhancing cell viability after exposure and down-regulating the UV-induced expression of *KRT1* and *KRT10* keratin genes responsible of skin photoaging via epidermal thickening and decrement of elasticity. When also considering the radical scavenging activity shown by these collagen hydrolysates and their lack of toxicity towards different cultured cytotypes, the peptide fractions from *C. reniformis* collagen show considerable biotechnological potential in the development of formulations that are designed to mitigate the damages in UV-exposed or wounded skin.

**Figure 3:**
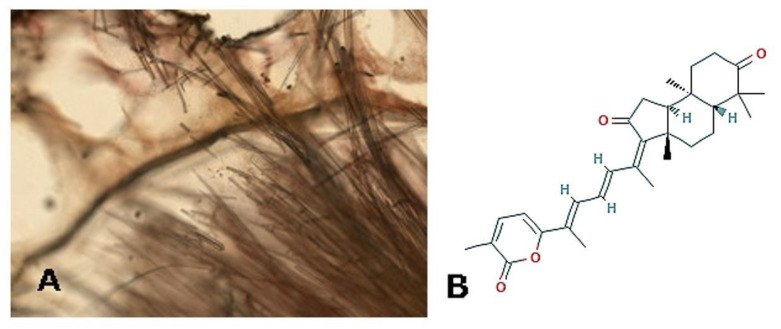
(**A**) Image of a paraffin section of *Jaspis stellifera* demosponge. Photo by D. Drew (YPM IZ 087028). Courtesy of the Peabody Museum of Natural History, Division of Vertebrate Paleontology, Yale University; peabody.yale.edu. (**B**) 2D structure of stellettin B; https://pubchem.ncbi.nlm.nih.gov/compound/5352082#section=2D-Structure.

*Jaspis stellifera* [Carter, 1879; Demospongiae, Tetractinellida: Ancorinidae; Figure 3A] is another sessile species typical of tropical and Indo-West Pacific environments. A number of anti-cancer bioactive molecules have been isolated from this marine invertebrate, such as jaspiferins, jaspiferals, stelliferins, and stellettin [34,35,36,37,38,39,40,41]. In their publication, Cheng et al. [42] produced additional data regarding the mobility control exercised by the triterpenoid stellettin B (3*Z*,3a*S*,5a*R*,9a*R*,9b*S*)-3a,6,6,9a-tetramethyl-3-[(3*E*,5*E*)-6-(5-methyl-6-oxopyran-2-yl)hepta-3,5-dien-2-ylidene]-4,5,5a,8,9,9b-hexahydro-1Hcyclopenta [a]naphthalene-2,7-dione; Figure 3B on human U87MG and GBM8401 glioblastoma cells. In particular, the migration and invasion of tumor cells was inhibited in both scratch wound healing and transwell chamber assays when exposed to an increasing concentration of the triterpene in the range between 1 and 10 μM. This appeared to be based, at least in part, upon the p-Akt downsignalling-mediated inhibition of the interaction between F-actin and the actin-binding protein P-girdin, responsible for pseudopoda extension at the cell leading edge, and the expression of matrix metalloprotease 2, phosphorylated integrin β1 and phosphorylated focal adhesion kinase by this cancer cytotype [43]. In consideration that glioblastoma cell survival and the angiogenetic processes in models in vitro and in vivo are also remarkably reduced in the presence of stellettin B [42], this molecule is a strong candidate as anti-cancer agent against malignant brain tumors.

**Figure 4:**
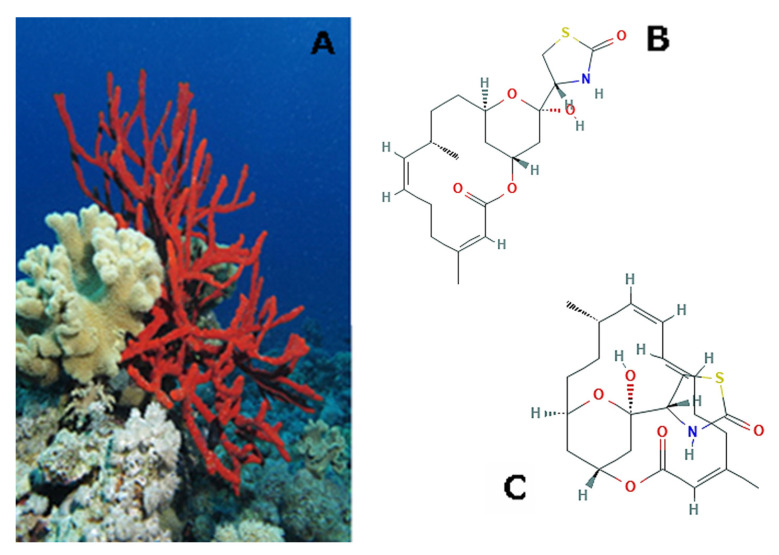
(**A**) A specimen of *Negombata magnifica* demosponge. Photo © Alexander Vasenin (CC BY-SA 3.0) https://en.wikipedia.org/. (**B**) Two-dimensional (2D) structure of latrunculin B; https://pubchem.ncbi.nlm.nih.gov/compound/Latrunculin-B#section=2D-Structure. (**C**) 2D structure of latrunculin A; https://pubchem.ncbi.nlm.nih.gov/compound/Latrunculin-a#section=2D-Structure.

*Negombata magnifica* (Keller, 1889; Demospongiae, Poecilosclerida: Podospongiidae), formerly named Latrunculia magnifica, is also known as toxic finger-sponge and it typically has a brilliant orange-red coloration and a digitate or leafy morphology with crooked branches (Figure 4A). It is distributed in the Indian Ocean and the Red Sea, where it intensively populates the coral reefs. When these sponges form colonies, they protect themselves via chemical defences, in particular releasing a reddish fluid containing latrunculins, toxic substances that are known to bind G-actin in a reversible manner and 1:1 stoichiometric ratio [44]. Latrunculin B (4*R*)-4-[(1*R*,4*Z*,8*Z*,10*S*,13*R*,15*R*)-15-hydroxy-5,10-dimethyl-3-oxo-2,14-dioxabicyclo[1 1.3.1] heptadeca-4,8-dien-15-yl]-1,3-thiazolidin-2-one; Figure 4B is a member of this family that is a promising agent for the development of anti-glaucoma and anti-arrythmia therapies [45,46]. This macrolide and its semi-synthetic derivatives 15-*O*-methyl-latrunculin B, *N*-hydroxymethyl- latrunculin B and *N*-acetyl-latrunculin B were found to restrain the migration of the murine brain-metastatic melanoma cell line B16B15b in both transwell chamber and scratch wound healing assays [47]. Latrunculin A ((4*R*)-4-[(1*R*,4*Z*,8*E*,10*Z*,12*S*,15*R*,17*R*)-17-hydroxy- 5,12-dimethyl-3-oxo-2,16dioxabicyclo[13.3.1] nonadeca-4,8,10-trien-17-yl]-1,3-thiazolidin-2-one; Figure 4C and its carbamate derivatives 17-*O*-[*N*-(phenyl)carbamoyl]-latrunculin A, 17-*O*-[*N*-(3-chloropropyl)carbamoyl] latrunculin A and 17-*O*-[*N*-(benzyl)carbamoyl]-latrunculin A appeared to be able to impair the invasive behavior of prostate cancer cells in Boyden chambers in the presence of Matrigel, as well as the disaggregation of spheroids in vitro [48]. In this paper, a putative inhibitory effect of these molecules on the activation and signalization of hypoxia-inducible factor (HIF)-1, a transcription factor that is involved in the metastatic attitude of prostate cancer cells [49], was presumed. Afterwards, the in vitro invasive phenotype of MDA-MB231 breast cancer cell was also found to be impaired in Boyden chamber assays by derivatives of latrunculin A, the most potent being 17-*O*-phenylethyl-latrunculin A and *N*-*p*-methoxyphenylacetyl-15-*O*-methyl-latrunculin A. Interestingly, the latter one is likely to recognize different and yet unknown target(s) than actin microfilaments. Of note, 15-*O*-methyl-latrunculin B also exhibited a powerful anti-angiogenic activity, whereas 17-*O*-phenylethyl-latrunculin A was significantly cytotoxic against MCF-7 breast cancer cells [50], with both properties being appropriate to potential anti-breast cancer drugs.

**Figure 5:**
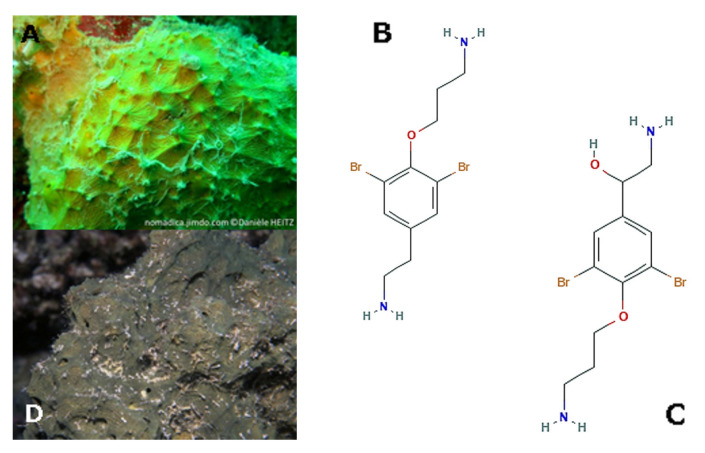
(**A**) A specimen of *Pseudoceratina arabica* demosponge © Danièle Heitz; https://nomadica.jimdofree.com/. (**B**) 2D structure of moloka’iamine; https://pubchem.ncbi.nlm.nih.gov/compound/Moloka_Iamine#section=Structures. (**C**) 2D structure of hydroxymoloka’iamine; https://pubchem.ncbi.nlm.nih.gov/compound/25016152. (**D**) A specimen of *Suberea mollis* demosponge; Abbas et al., 2014 (CC BY 3.0).

*Pseudoceratina arabica* (Keller, 1889; Demospongiae, Verongiida: Pseudoceratinidae; Figure 5A is distributed in the shallow sandy lagoons and seagrass beds of the southern Red Sea. It appears as a sprawling and branching sponge and his body might show varying characteristics, appearing dense, rubbery, flexible, or waxy. A number of literature data indicate that these sponges produce several compounds with varied biological activities, e.g., the antimicrobial and antifungal parasympatholytic moloka’iamine and moloka’iakitamide, and the skeletal α-chitin of particular interest for different biotechnological applications, such as electrochemistry and regenerative medicine [51,52]. The bromotyrosine derivative (1’*R*,5’*S*,6’*S*)-2-(3’,5’-dibromo-1’,6’ -dihydroxy-4’-oxocyclohex-2’-enyl) acetonitrile obtained from *P. arabica* was found to impair the TGFβ-stimulated epithelial-mesenchymal transition of A549 lung cancer cells and the related cell migration in scratch wound closure assays through the inhibition of both the activity of type I TGF-β receptor kinase ALK5 and the consequent motility-addressed actin microfilament reorganization thereby being a promising anti-tumoral pharmacological tool [53]. Noteworthy, the bidimensional locomotion of the highly-malignant MDA-MB231 breast cancer cells in wound closure assay was consistently restrained by the already-mentioned moloka’iamine (3-[4-(2-aminoethyl)-2,6- dibromophenoxy]propan-1-amine; Figure 5B and its *P. arabica*-obtained derivative ceratinin A, whereas the in vitro invasive activity of the same cell line in transwell assays was decreased by exposure to two other P. arabica-obtained moloka’iamine derivatives, i.e., ceratinin D and hydroxymoloka’iamine (2-amino-1- [4-(3-aminopropoxy)-3,5- dibromophenyl] ethanol; Figure 5C. In the same paper, evidence was shown that also the alkaloid subereamolline A produced by another verongid sponge distributed in the Southern Red and Western Arabian Sea, i.e., *Suberea mollis* (Row, 1911; Demospongiae, Verongiida: Aplysinellidae; Figure 5D) was able to inhibit the scratch wound healing in vitro by MDA-MB231 breast cancer cells [54]. Of note, *S. mollis* is a good producer of a number of compounds with overt anti-microbial and anti-oxidant hepatoprotective functions, such as aeroplysinins, the brominated arginine-derived alkaloids subereamines, and the brominated phenolic molecules subereaphenols, among others [55,56,57].

**Figure 6:**
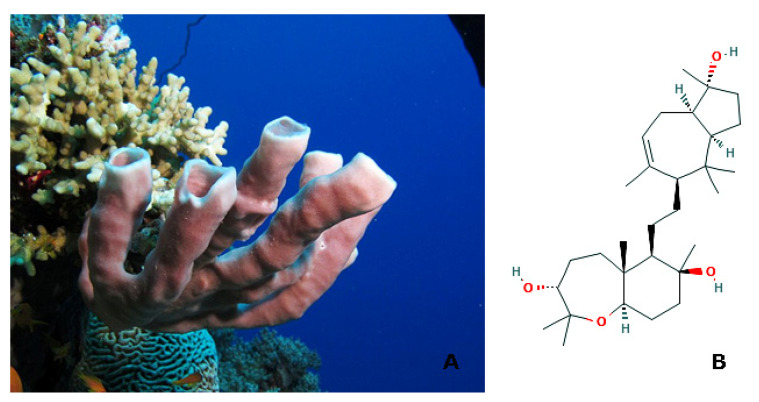
(**A**) A specimen of *Siphonocalina siphonella* demosponge © Alexander Vasenin (CC BY-SA 3.0); https://commons.wikimedia.org/. (**B**) 2D structure of sipholenol A; https://pubchem.ncbi.nlm.nih.gov/compound/Sipholenol-A#section=Structures.

*Siphonocalina* (*Callyspongia*) *siphonella* (Lévi, 1965; Demospongiae, Haplosclerida: Callyspongiidae; Figure 6A), also named colonial tube-sponge for its appearance as a bunch of pale lavender long tubes, is a species typical of the Red Sea. This organism is a source of secondary metabolites endowed with anti-infective and anti-inflammatory properties, and cytotoxicity against cancer cells [58,59,60]. Sipholenol A ((3*R*,5a*R*,6*R*, 7*S*,9a*R*)-6- [2-[(1*R*,3a*R*,5S,8a*R*)-1-hydroxy-1,4,4,6-tetramethyl-2,3,3a,5,8,8a-hexahydroazulen-5-yl]ethyl]-2,2,5a,7-tetramethyl-4,5,6,8,9,9a-hexahydro-3H-benzo[b]oxepine-3,7-diol; Figure 6B is a triterpenoid that is produced by this demosponge displaying an anti-cancer activity that is based upon its ability to counteract the multidrug resistance of neoplastic cells via direct inhibition of P-glycoprotein [61]. Aliphatic polar and aromatic ester derivatives of this molecules exhibited a strong anti-migratory and anti-invasive effect towards MDA-MB231 malignant breast cells in scratch wound healing and Boyden chamber assay in the presence of basement membrane extract [62]. The down- regulation of cell motility appeared to be due, at least in part, to the inhibition of phosphorylation of protein tyrosine kinase-6 (PTK6), an anti-apoptotic and pro-metastatic factor for this cell line [63]. Akl et al. [64] demonstrated that the sipholenol A-4-*O*-3′,4′-dichlorobenzoate derivative was also active as potential anti-breast cancer compound. In particular, they found that this molecule inhibited the proliferation of a panel of breast tumor cell lines with different neoplastic phenotypes, impairing cell cycle progression, and it restrained both cell motility in transwell chamber assays in vitro and tumor mass expansion in MDA-MB231 xenograft nude mice model in vivo. Such effects appeared to be based, at least in part, upon the interference with the metastasis-associated breast cancer kinase (BRK) and focal adhesion kinase (FAK) signal cascades, in turn inhibiting the activation of some downstream signalling factors, such as AKT, mitogen-activated protein kinase (MAPK), and paxillin. Thus, sipholenol A appears to be a promising scaffold for the development of novel anti-malignant breast cancer drugs.

**Figure 7:**
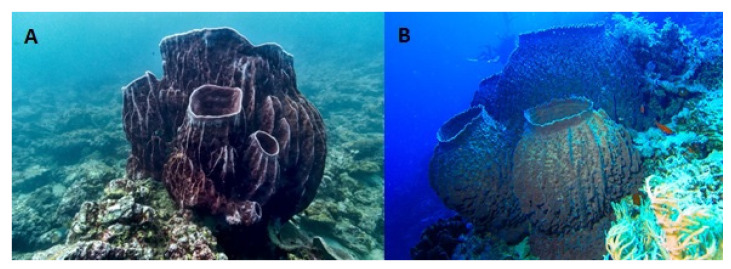
Examples of specimens of *Xestospongia* sp. (**A**) *X. muta* © Renaud Houdinet; https://flickr.com/; (**B**) *X. testudinaria*, photo by Albert Kok (Public domain) https://commons.wikimedia.org/.

*Xestospongia* sp. (Demospongiae, Haplosclerida: Petrosiidae; Figure 7 is widely distributed in the Indian and South Pacific Oceans. These animals represent a rich source of secondary metabolites with very different biological properties that have been shown effective as enzymatic inhibitors, anti-malarial, anti-bacterial, vasodilatory, and anti-cancer agents [65,66,67,68,69,70]. Among them, ranieramycins are a group of biologically-active tetrahydroisoquinoline compounds. Halim et al. [71] reported a significant inhibitory effect exerted by a member of this family, ranieramycin M, on the motile and invasive behavior of H460 lung cancer cells in scratch wound healing and matrigel-containing transwell assays at non-toxic or growth-impairing concentrations.

## 3. Cnidaria

**Figure 8:**
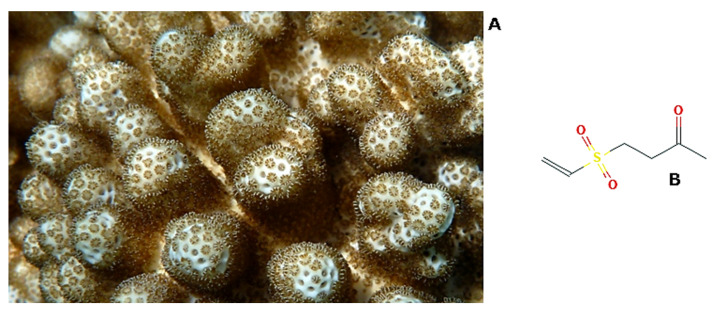
(**A**) A specimen of *Cladiella australis* soft coral © David (CC BY-NC-SA 4.0) https://www.marinespecies.org/. (**B**) 2D structure of austrasulfone; https://pubchem.ncbi.nlm.nih.gov/compound/50907657#section=Structures.

*Cladiella australis* (Macfadyen, 1936; Anthozoa, Alcyonacea: Alcyoniidae; Figure 8A), also known as the finger blanching soft coral, is a species distributed in the Western Pacific ocean, displaying brown or white color, depending on whether the polyps are extended or retracted. It is know that this organism produces several secondary metabolites with promising biological activities as hypoglicemic, anti-cancer and anti-melanogenic compounds, the latter having potential applications in medical cosmetology [72,73,74]. Among the molecules that were obtained from *C. australis*, research interest has been focused on the neuroprotective austrasulfone (4-ethenylsulfonylbutan-2-one; Figure 8B [75]. Its synthetic precursor, dihydroaustrasulfone alcohol, exhibited the ability to restrain the migratory behaviour of A549 lung tumor cells in both wound healing and transwell chamber assays in vitro. This inhibitory activity appeared to be the result of the inactivation of extracellular signal-regulated kinase (ERK) 1/2 intracellular signalling, which, in turn, down-regulated the levels of activated focal adhesion kinase (FAK), PI3K, and p-AKT. Such derangement of transduction mechanisms also determined the decrease of the production of matrix metalloproteinase (MMP)-2 and -9 enzymes, responsible for the observed reduced tumor size in a A549 xenograft nude mice model [76]. Interestingly, dihydroaustrasulfone alcohol was also proven to restrain the platelet-derived growth factor (PDGF)-BB promoted the proliferation and migration of vascular smooth muscle cells, as documented by wound healing, Boyden chamber, and transwell assays, thereby playing a vasoprotective role and being a good candidate for the treatment of vascular occlusive diseases [77,78].

**Figure 9:**
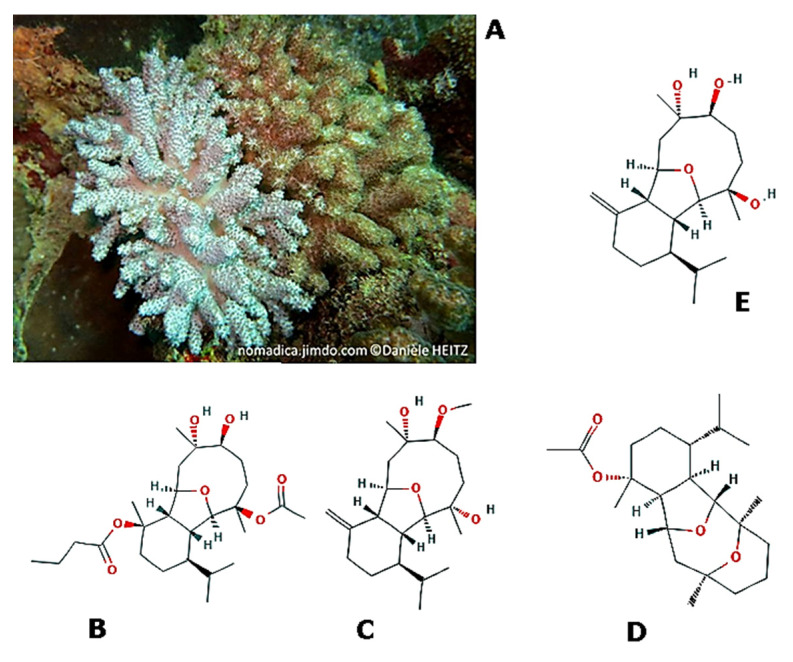
(**A**) A specimen of *Cladiella pachyclados* soft coral © Danièle Heitz; https://nomadica.jimdofree.com/. (**B**) Two-dimensional (2D) structure of pachycladin A; https://pubchem.ncbi.nlm.nih.gov/compound/Pachycladin-A#section=2D-Structure. (**C**) 2D structure of sclerophytin F methyl ether; https://pubchem.ncbi.nlm.nih.govcompound/21635655. (**D**) 2D structure of polyanthelin A; https://pubchem.ncbi.nlm.nih.gov/compound/12168107. (**E**) 2D structure of sclerophytin A; https://pubchem.ncbi.nlm.nih.gov/compound/Sclerophytin-A#section=2D-Structure.

*Cladiella pachyclados* (Klunzinger, 1877; Anthozoa, Alcyonacea: Alcyoniidae; Figure 9A) is a soft coral typical of reef-associated and subtropical, in particular Western Pacific, environments. It is known that this marine invertebrate produces eunicellin (i.e., [(1*R*,2*R*,3*R*,5*S*,6*S*,7*S*,8*R*,11*S*,14*R*)-5,6,14-triacetyloxy-6, 14-dimethyl-10-methylidene-3-propan-2- yl-15-oxatricyclo[6.6.1.02,7]pentadecan-11-yl]acetate)-based diterpenes with anti-inflammatory and anti-cancer properties. In particular, Hassan et al. [79] showed the powerful anti-migratory and anti-invasive role played in vitro on PC3 prostate cancer cells by some of these compounds, with the most effective in both experimental tests being pachycladin A ([(1*R*,2*S*,3*R*,6*R*,7*R*,8*R*,9*R*,12*S*,13*S*)-9-acetyloxy-12,13-dihydroxy-3,9,13- trimethyl-6-propan-2-yl- 15 oxatri cyclo[6.6.1.02,7]pentadecan-3-yl] butanoate; Figure 9B, sclerophytin F methyl ether ((*1R,2R,6R*,*7R,8R,9S,12S,13S*)-12-methoxy-9,13-dimethyl-3- methylidene-6-propan-2-yl-15 oxatricyclo[6.6.1.02,7]pentadecane-9,13-diol; Figure 9C), polyanthelin A ([(*1R,2R,6S,8R,9S,10R,13R,14R*)-2,6,10-trimethyl-13-propan-2-yl- 15,16- dioxatetracyclo [6.6.1.12,6.09,14]hexadecan-10-yl] acetate; Figure 9D, and sclerophytin A ((*1R,2R,6R,7R,8R,9R,12S,13S*)-9,13-dimethyl-3-methylidene-6-propan-2-yl-15oxatricyclo[6.6.1.02,7] pentadecane-9,12,13-triol; Figure 9E [79].

**Figure 10:**
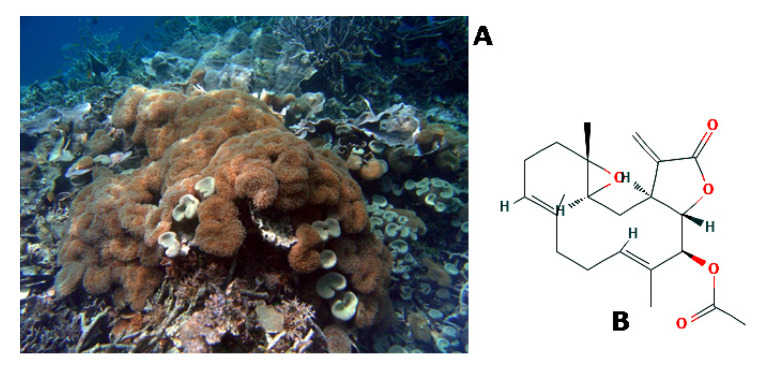
(**A**) A specimen of *Sarcophyton crassocaule* soft coral © Leen Van Ofwegen (CC BY-NC-SA 4.0); https://www.marinespecies.org/ (**B**) 2D structure of 13-acetoxysarcocrassolide; https://pubchem.ncbi.nlm.nih.gov/compound/21775908.

*Sarcophyton crassocaule* (Moser, 1919, Anthozoa, Alcyonacea: Alcyoniidae; Figure 10A is a subtropical species of sessile soft corals distributed in the Western Pacific area, New Caledonia, Taiwan and Ryukyu Island. A number of terpenoids with anti-tumoral properties have been extracted by this marine invertebrate [80]. Studies in vitro on the anti-migratory effects of compounds that are produced by S. crassocaule have been focussed on bladder and gastric tumor cell models. In the first case, the cembrenolide diterpene 13-acetoxysarcocrassolide ([(*1S,3S,5S,8E,12E,14S,15S*)-5,9,13- trimethyl-18-methylidene-17-oxo-4,16-dioxatricyclo[13.3.0.03, 5]octadeca-8,12-dien-14-yl] acetate; Figure 10B) was proven to impair locomotion in scratch wound healing assays, apart from inducing apoptotic cell death after 24 h of exposure. Western blot and proteomic analyses demonstrated the up- or down-regulation of a number of proteins, including members of the group of heat shock proteins, key enzymes of the Krebs cycle, and proteins that are associated to apoptosis and DNA repair, although no correlation between the biochemical data and the anti-migratory effect was shown [81]. Similar results on 13- acetoxysarcocrassolide-induced motility restraining were obtained with gastric carcinoma cells, in which the up-regulation of p38- and c-Jun N-terminal kinase (JNK)-transduction pathways and down-regulation of phosphoinositide 3-kinase (PI3K)/AKT signalization were also observed [82].

**Figure 11:**
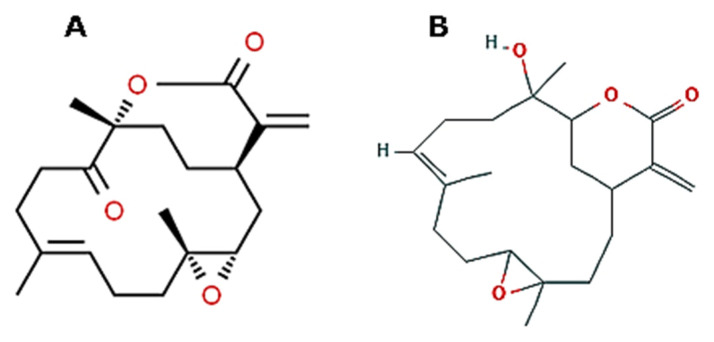
(**A**) 2D structure of 11-dehydrosinulariolide; http://www.chemspider.com/Chemical-Structure.10470882.html. (**B**) 2D structure of sinularin; https://pubchem.ncbi.nlm.nih.gov/compound/Sinularin#section=2D-Structure.

*Sinularia nanolobata* (Verseveldt, 1977; Anthozoa, Alcyonacea: Alcyoniidae) populates the coral reefs or rocks in shallows from East Africa to the Western Pacific area. The bioactive compound 11-dehydrosinulariolide (*1R,3S,5S,8E,13R*)-5,9,13-trimethyl-16-methylene-4,14-dioxa tricyclo[11.3.2.03,5] octadec-8-ene-12,15-dione11-dehydrosinulariolide; Figure 11A that is produced by this soft coral has been tested for the control of the migratory behavior of CAL-27 oral squamous cancer cells in scratch wound healing assays. The obtained results demonstrate that the wound repair function is down-regulated by the molecule, as well as cell growth and survival, whereas apoptotic death is promoted. Proteomic analyses showed that exposure to 11-dehydrosinulariolide determined the modification of the levels of proteins that are associated with cell growth, apoptosis, translation and protein folding, and energy metabolism [83]. Subsequent investigations on another bioactive molecule extracted from S. nanolobata, i.e., sinularin ((9*E*)-13-hydroxy-4,9,13-trimethyl-17-methylidene-5,15-dioxatricyclo[12.3.1.04,6] octadec-9-en-16-one; Figure 11B), confirmed this compound as an additional potential anti-cancer molecule. In fact, sinularin promoted the inhibition of growth and motility, the block at G_2_/M cell cycle phase, and the onset of apoptosis in A2058 melanoma cells and proteomic analyses found modifications in the levels of oncosuppressor and apoptosis-associated proteins consistent with the observed phenotypic changes [84].

**Figure 12:**
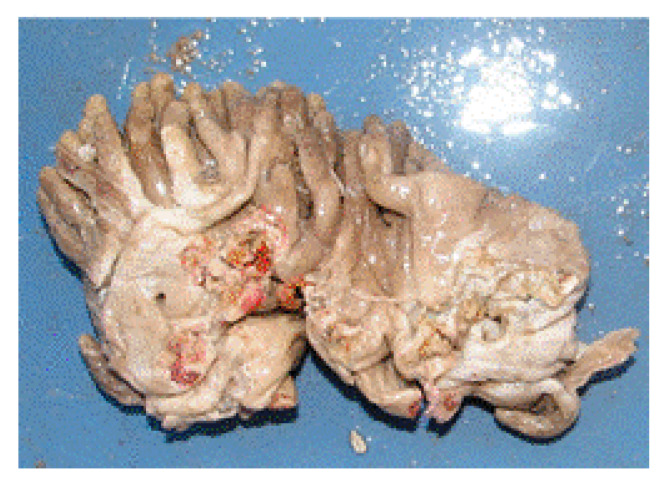
An image of *Clavularia koellikeri*; Wang et al. (2019) [87].

*Clavularia koellikeri* (Dean, 1927; Anthozoa, Alcyonacea, Clavulariidae; Figure 12) is a widespread species throughout the Indian and Pacific oceans, from the Red Sea and East Africa across the Indo-Pacific to Micronesia, being a typical inhabitant of the Great Barrier Reef. Soft corals belonging to this taxonomic genus are an important resource of secondary metabolites, mainly terpenoids, which are endowed with unique structures and a wide range of biological activities [85,86]. Among them, clavukoellian A and clavukoellian D-related 4-*O*-deacetylparalemnolin D exerted an anti-locomotory effect on human umbilical vein endothelial (HUVEC) cells in wound healing assays, thus showing interesting anti-angiogenetic activity that merits further investigation [87].

**Figure 13:**
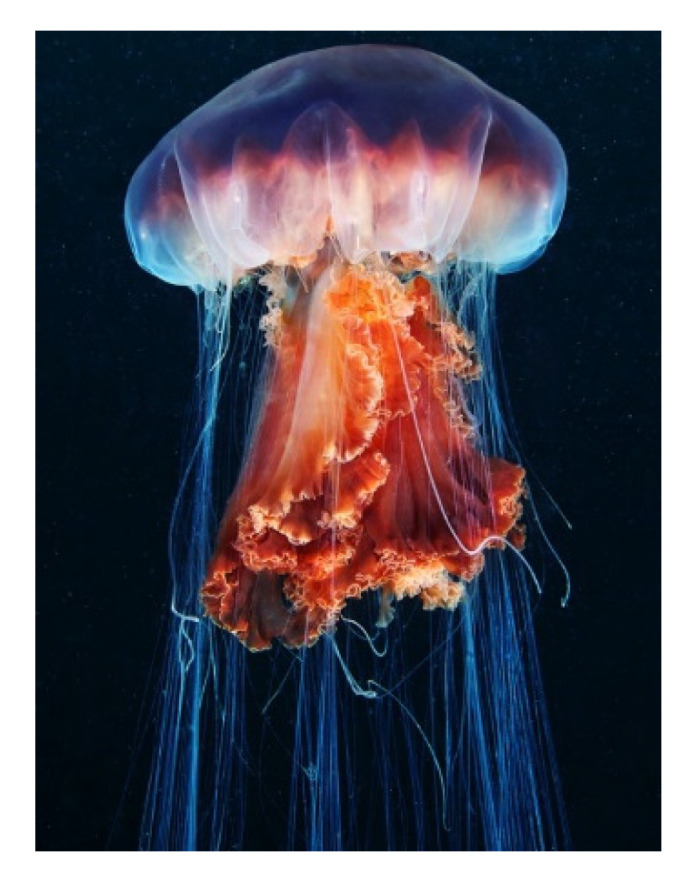
An indivividual of *Cyanea capillata* © Alexander Semenov; https://www.flickr.com/.

*Cyanea capillata* (Linnaeus, 1758; Scyphozoa, Semaeostomeae: Cyaneidae; Figure 13), commonly known as lion’s mane jellyfish, populates the cold waters of the more northerly areas of the North and Arctic seas and the Pacific and Atlantic oceans, also finding itself along the coasts of Southeast China [88,89,90]. This organism represents the largest jellyfish known to date, with an umbrella from which originate a large number of reddish-brown or yellowish tentacles long up to 30 m and wide up to one meter, endowed with considerable amounts of nematocysts that release toxins dangerous for humans [91]. It is widely acknowledged that jellyfishes are important sources of many different bioactive molecules. This species, in particular, is a good producer of antioxidants conceivably aimed to protect its supercoiled DNA from oxidative damage. In fact, the molecular cloning and recombinant protein production of peroxiredoxin-, thioredoxin-, and Cu/Zn superoxide dismutase (SOD) homologues was successfully performed starting from cDNA libraries of *C. capillata* tentacles [92,93,94]. In addition, while using the same molecular source a recombinant protein with potent anti-bacterial activity was expressed and purified [95]. Wang et al. [96] demonstrated the promoting effect of *C. capillata*’s tentacle extracts on the migratory behavior of human umbilical vein endothelial cells in scratch wound healing assays, thus considering this mixture a good candidate as wound healing regulator, improving tissue repair in unfavorable conditions. In a following study, they isolated and purified a *C. capillata*-specific granulin, the 5782 Da-bioactive component of the extract, able to trigger cell locomotion, and also up-regulation of cyclin B1 and D1 and cell growth, via the activation of ERK1/2 and MAPK signal cascade [97].

**Figure 14:**
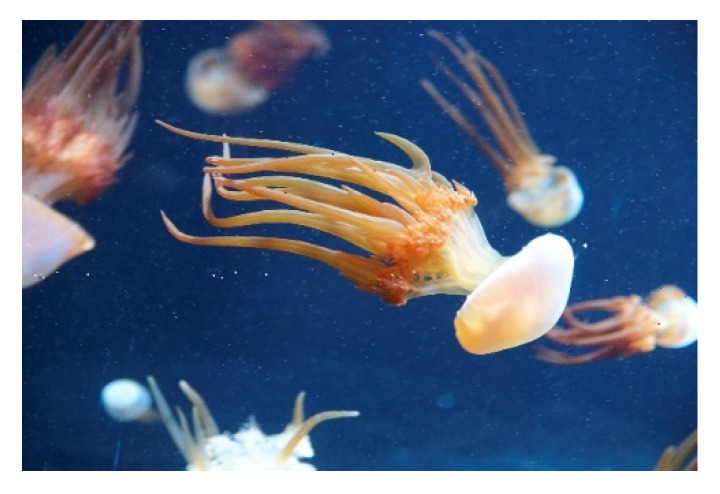
A specimen of *Rhopilema esculentum*. © Bill Abbott (CC BY-SA 2.0); https://en.wikipedia.org/.

*Rhopilema esculentum* (Kishinouye, 1891; Scyphozoa, Rhizostomeae: Rhizostomatidae; Figure 14) is a jellyfish species that inhabits pelagic and tropical environments, in particular populating the Northwestern Pacific area, the coasts of China and Japan, the Yellow Sea and the Bohai Sea. It can achieve the diameter of several cm and the weight of 30 kg. This organism is commonly known as flame jellyfish due to its mouth-arms that are endowed with abundant filamentous and large spindle-shaped appendices bearing undefined terminal clubs. *R. esculentum* is an eatable species, widespread in the market for its pleasant flavour and its lipid profile rich of fatty acids and glycerophospholipids that makes it a potential healthy food for human consumption and an aliment that is suitable for some commercial fishes and crustaceans [98,99]. In addition, significant insecticidal properties were demonstrated in the full proteinous venom of this invertebrate, thereby being a potential component of environmentally-friendly insecticides [100], whereas peptides that were obtained from *R. esculentum*’s hydrolysates exhibited angiotensin I-converting enzyme (ACE)-inhibitory and, therefore, anti-hypertensive and anti-oxidant properties [101,102]. Research interest has recently been focused on the tissue repair properties of the collagen component extracted from this organism. Felician et al. [103] demonstrated the ability of preparations of collagen peptides, which were obtained through either collagenase II- or papain digestion, to induce the motility of human vein umbilical cells in scratch wound healing assays. Moreover, the same preparations were applied to the back of wounded mice and the results that were obtained in vivo confirmed their regenerative properties determining a significant decrease of the wounded area, especially in case of treatment with collagenase II-digested samples. Immunohistochemical analyses of the areas of the in-progress tissue reconstitution showed the up-regulation of TGFβ1 and β-FGF, known factors that are involved in the inflammatory and angiogenetic phases of skin repair whose acceleration by the peptide mixture makes it a promising product for wound repair. Noteworthy, the collagen component extracted from *R. esculentum* has also been the object of studies showing its remarkable anti-photoaging and hemostatic properties, and its suitability and safety for use as a scaffold supporting the chondrogenic differentiation of adult stem cells in cartilage tissue engineering, which merits further exploitation [104,105,106].

## 4. Mollusca

**Figure 15:**
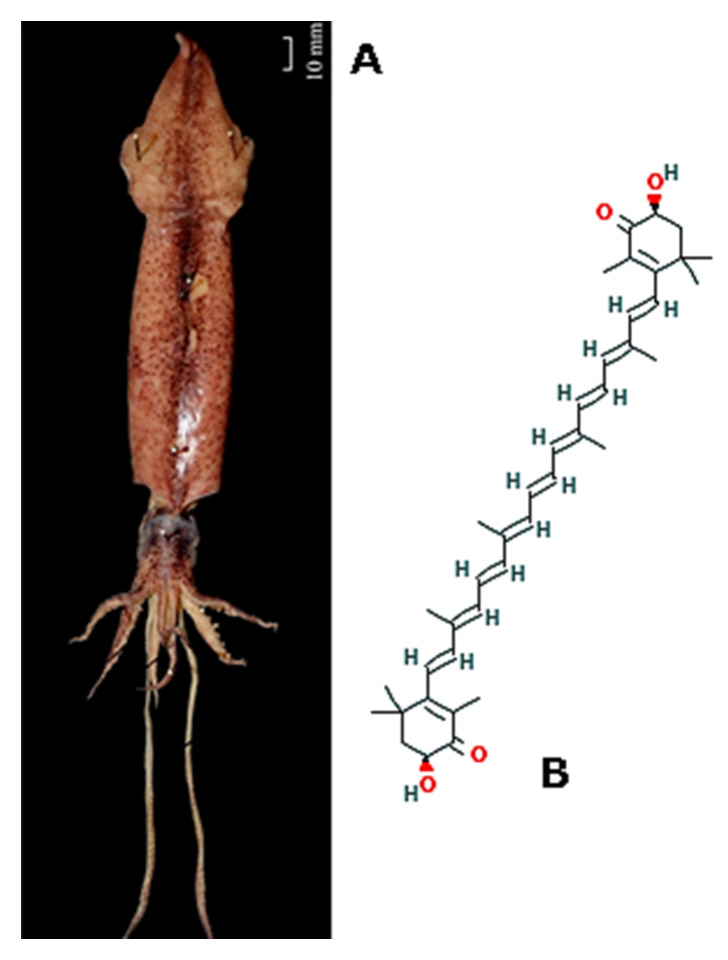
(**A**) A specimen of *Uroteuthis singhalensis*; http://www.borneomolluscs.com. (**B**) 2D structure of astaxanthin; https://pubchem.ncbi.nlm.nih.gov/compound/Astaxanthin#section=2D-Structure.

*Uroteuthis* (*Doryteuthis*) *singhalensis* (Ortmann, 1891; Cephalopoda, Myopsida: Loliginidae, Figure 15A) is a demersal species, which is typical of subtropical and tropical areas, especially of the Indo-West Pacific zone, representing one of the commercially-important squid species in India. This cephalopod has been used as a source of the polysaccharide chitosan, which exhibits powerful antioxidant and anticoagulant properties, and of invertebrate collagen, which is a good alternative to the mammalian counterpart being the animal’s skin available in large amount as fish markets’ left-over [107,108]. Veeruraj et al. [109] recently reported the application of a biocomposite matrix that is constituted by the radical scavenging-carotenoid astaxhantin ((6*S*)-6-hydroxy-3-[(*1E,3E,5E,7E,9E,11E,13E,15E,17E*)-18-[(4*S*)-4-hydroxy-2,6,6- trimethyl-3- oxocyclohexen-1-yl]3,7,,12,16-tetramethyloctadeca-1,3,5,7,9,11,13,15,17-nonaenyl]-2,4,4-trimethylcyclo hex-2-en-1-one; Figure 15B incorporated into a scaffold of collagen, both extracted from U. singhalensis, for the healing of dermal wounds of rats in vivo. The obtained data demonstrated a higher wound contraction rate and tensile strenght and a faster epithelization time at the excision site, with an increased deposition of newly synthesized collagen in the damaged area following fibroblast proliferation and activation. Histopathological observations confirmed the remarkable healing properties of the material, additionally putting the onset of a pronounced and accelerated revascularization of the tissue in evidence. The obtained cumulative results highlighted the potentiality of this composite sponge in providing a superior-quality wound dressing, also in terms of stability, low-cost, biodegradability, and biocompatibility, which may have additional applications in tissue engineering and regenerative medicine.

**Figure 16:**
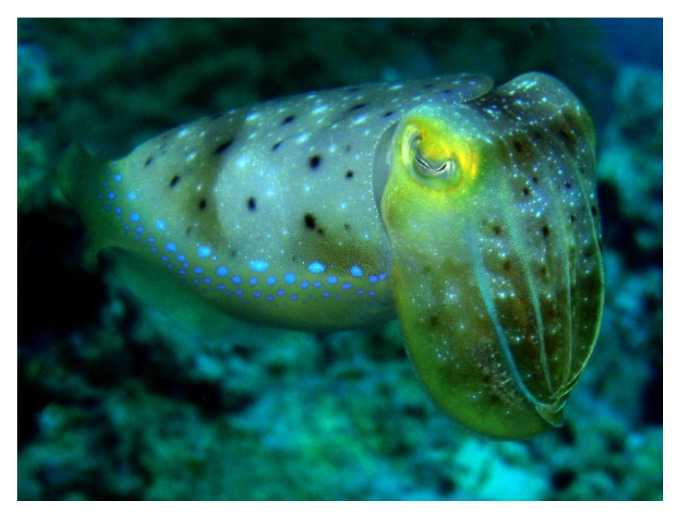
A specimen of *Sepia kobiensis*. © Maynard Hogg (CC BY-NC-SA 2.0); https://www.flickr.com/.

*Sepia kobiensis* (Hoyle, 1885; Cephalopoda, Sepiida: Sepiidae; Figure 16), also known as kobi cuttlefish, is a benthic species distributed in the Indo-West Pacific area from Japan to the Persian Gulf. It is an eatable organism with a nutritional value that is very close to that of fin fish, although the various parameters tested (i.e., protein, moisture and ash contents, and total lipid, cholesterol and triglyceride levels) vary in the different parts of the body, head, arm, tentacle, and coat [110]. Additionally, chitosan and collagen of this cephalopod attracted keen research interest. The first component showed a number of therapeutically-interesting properties, i.e., anti-bacterial and anti-microbial, as well as anti-oxidant and anti-lipidemic activities, the latter being successfully applied to in vivo rat models of carbon tetrachloride-induced hepatic damage [111,112,113,114]. Ramasamy and Shanmugan [115] prepared a bioscaffold with collagen and chitosan extracted from S. kobiensis and tested it in an in vivo rat wound healing model. They found that the hybrid film determined an increased wound reduction and fibroblast proliferation and differentiation towards myofibroblasts, thereby determining the up-regulation of collagen deposition in the damaged tissue. Moreover, the presence of chitosan also guaranteed a free radical-scavenging action that protected the proliferating cytotypes, i.e., epithelial cells and fibroblasts, from the cytotoxic influence of reactive oxygen species (ROS). Thus, the merging of the beneficial effects of the two macromolecules makes this material a promising candidate as a dressing that supports a faster wound repair process.

**Figure 17:**
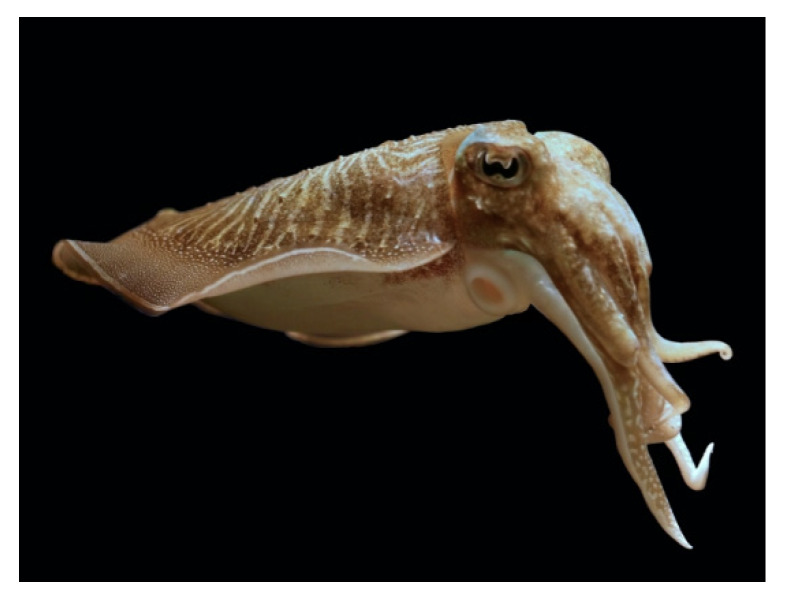
A specimen of *Sepia officinalis*. © Hans Hillewaert (CC BY-SA 4.0); http://www.marinespecies.org/.

Successful results were also achieved utilizing collagen preparations from the local cuttlefish *Sepia officinalis* (Linnaeus, 1758; Cephalopoda, Sepiida: Sepiidae; Figure 17), an invertebrate that is widely distributed in the Western Mediterranean Sea, which can be also found along the West African coasts. Collagen hydrogels were applied to the excised skin of female Wistar rats and proven to be a good quality dressing promoting tissue restoration to levels that are comparable to those of a commercial vegetable extract-based emulsion used as reference. In particular, *S. officinalis*’s collagen films induced a quick contraction and closure of the wound, with an absence of inflammation signs and, on the other hand, evidence of neo-vessel growth and active ECM deposition at the histological analysis [116]. Of note, collagen preparations of this cephalopod are widely used for diverse application, such as gelling agent, thickener, foaming agent, and emulsifier in food industries or natural bioactive filler promoting implant osteointegration [117,118].

**Figure 18:**
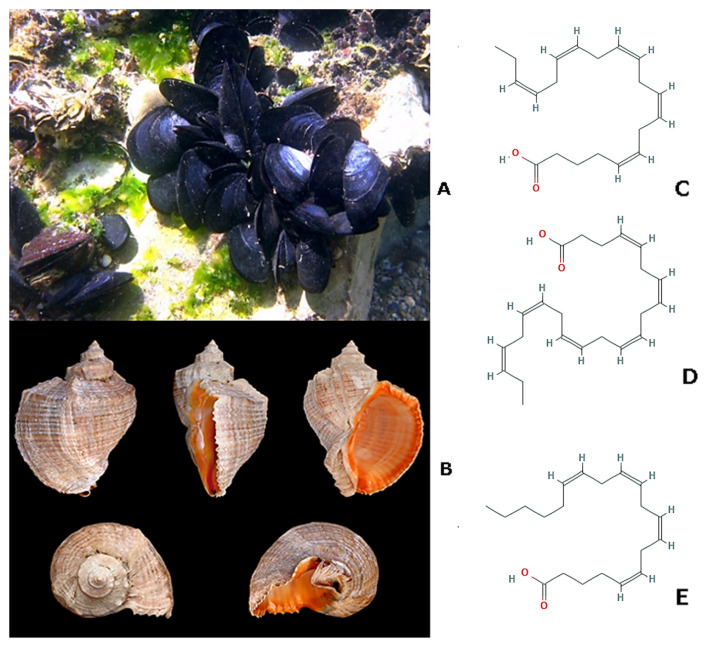
(**A**) A group of *Mytilus galloprovincialis* bivalves. © Pillon Robert (CC BY-NC-SA 4.0); http://www.marinespecies.org/. (**B**) Images of the shell of *Rapana venosa*. © H. Zell (CC BY-SA 3.0); https://en.wikipedia.org/. (**C**) 2D structure of eicosapentaenoic acid; https://pubchem.ncbi.nlm.nih.gov/compound/Eicosapentaenoic-acid#section=2D-Structure. (**D**) 2D structure of docosahexenoic acid; https://pubchem.ncbi.nlm.nih.gov/compound/Docosahexaenoic-acid#section=Structures. (**E**) 2D structure of arachidonic acid; https://pubchem.ncbi.nlm.nih.gov/compound/Arachidonic-acid#section=2D-Structure.

*Mytilus galloprovincialis* (Lamarck, 1819; Bivalvia, Mytilida: Mytilidae; Figure 18A) is a widely distributed bivalve mollusk populating the Mediterranean and the Black Sea, the eastern Atlantic ocean from Morocco to the British Isles, but also present in Japan, California, South Africa, southern Australia, and New Zealand. It is a lamellibranch mollusk, which is, equipped with lamellar gills that absorb oxygen and simultaneously retain food for feeding. Therefore, being a filtering organism, it plays an indispensable role at ecological level, as marine purifier and it is widely used to evaluate the effects of stress environmental condition [119,120]. These invertebrates are endowed with an excellent nutritional value, owing to their remarkable richness in proteins, carbohydrates, essential vitamins, minerals, and different varieties of fatty acids that play beneficial roles for human health and, for this reason, they are in great demand on the international market [121,122,123]. *Rapana venosa* (Valenciennes, 1846; Gastropoda, Neogastropoda: Muricidae; Figure 18B) is a predatory benthic species of tropical environments typical of the Northwest Pacific area and introduced in the Atlantic, Mediterranean, and Black Sea [124,125]. It shows a variable color from grey to orange-brown or blonde, with darker brown dashes on the spiral ribs, and it is a widely consumed and commercially valuable mollusk in China. Recently, considerable interest has been paid to the concentrations of metals or pollutants, even radioactive, which *R. venosa* might contain, for an assessment of environmental monitoring and also the protection of human health, since this species is eatable and frequently consumed [126,127]. The anti-viral, anti-bacterial, and anti-cancer potentials of the hemocyanins obtained from the snail hemolymph have been extensively analyzed and demonstrated, thereby being promising components for formulations of anti-microbial and anti-tumoral vaccines [128,129,130]. Moreover, *R. venosa*’s shell was utilized to fabricate bionic coupling-layered ceramic/Al composites with improved lightweight and mechanical strength properties [131]. Being the gastropod a common predator of the mollusk *M. galloprovincialis*, Badiu and coworkers [132,133] examined in parallel the effect of lipid extracts and aminoacid preparations from *M. galloprovincialis* and *R. venosa* in in vivo wound healing tests.

Lipid extracts, containing slightly different complex mixtures with a high cholesterol content and good although varying amounts of several saturated and polyunsaturated fatty acids (PUFAs), including ω-3 eicosapentaenoic ((*5Z,8Z,11Z,14Z,17Z*)-icosa-5,8,11,14,17- pentaenoic acid, Figure 18C) and docosahexenoic acids, ((*4Z,7Z,10Z,13Z,16Z,19Z*)-docosa- 4,7,10,13,16,19-hexaenoic acid, Figure 18D) and, only for *R.venosa*, ω-6 arachidonic acid ((*5Z,8Z,11Z,14Z*)-icosa-5,8,11,14-tetraenoic acid, Figure 18E), were applied to skin burns in in vivo rat models in order to examine their potential healing activity. The results showed an acceleration of healing time when compared to a commercial fish oil-containing ointment used as reference, with complete re-epithelization and the deposition of dense ECM, re-vascularization, and absence of edema and inflammation Preparations from *M. galloprovincialis* exhibited a slightly faster recovery, thus suggesting the more active involvement of eicosapentanoic and docosahexenoic acids, which are abundant in its extract, in the wound healing process [132]. The same experimental study was also performed after application of aminoacid mixtures from protein extracts of *M. galloprovincialis* and *R. venosa*, which showed significant differences regarding aminoacidic distribution and content. Additionally, in this case, the correct formation of new epithelium and well-structured vessels, the improved collagen deposition and the reduced burn edema and leukocyte infiltration were observed [133]. This is likely due to the preponderance of thiol-containing aminoacids in the mixture, which exert anti-oxidant action preventing excessive lipid peroxidation and other aspects of the oxidative stress that are linked to the healing process. Of note, the damaged rat tissues healed faster when treated with *R. venosa*’s extracts, probably because of the beneficial effects of Leu, Lys, Thr, and Pro essential aminoacids, only present in these preparations.

**Figure 19:**
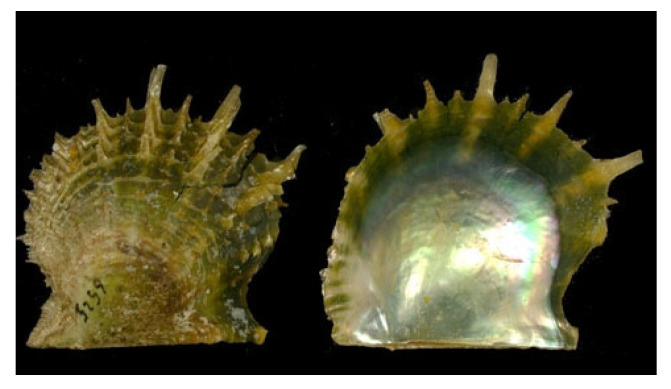
Images of the shell of *Pinctada imbricata*. © Natural History Museum Rotterdam (CC BY-NC-SA 4.0); http://www.marinespecies.org/.

*Pinctada martensii* (Dunker, 1880; Bivalvia, Ostreida: Margaritidae; Figure 19), also known as *Pinctada imbricata* (Röding, 1798), is a mollusk that is characteristic of benthic environments in the Atlantic and Indo-West Pacific areas and in the intertidal rocky regions of the Mumbai coast, to date listed among the most invasive species in the Mediterranean Sea [134]. It is one of the most important bivalves for the production of marine pearls all over the world and especially of the round «South China Sea pearls» that are typical of southern China. Despite the remarkable performance observed in the 90s [135], the rearing of this species subsequently decreased also due to environmental deterioration [136]. For this reason, this organism is commonly used to study the levels of environmental pollution, the effects of pollutants on the health of marine organisms, and the influence of various environmental stressors, such as changes in water temperature due to climate modification, on aquaculture yields. Proteins extracted from the meat and the nacre of this invertebrate have been shown to exert various functions, such as anti-microbial, anti-hypertensive, and osteogenetic [137,138,139]. Yang et al. [140] studied the effect of preparations of small molecular peptides from *P. martensii*’s mantle, i.e., the shell-producing organ, on skin wound repair in in vivo rat models. They demonstrated that the treatment was able to promote robust collagen deposition and re-epithelialization, thus accelerating wound healing, via the up-regulation of EGF, TGF-β1, and cyclin D1. The increase of CD31^+^ cytotypes in the tissue was indicative of enhanced neovascularization, whereas the up-regulation of IL-10 and the down-regulation of IL-6 was a sign of inhibition of the inflammatory response. A hypothetical explanation of the observed effect was based upon the high content in glutamic acid, glycine, cysteine, and phenylalanine of the preparations, which guarantee the nitrogen balance that is necessary for the continuous protein synthesis during angiogenesis, fibroblast growth, collagen deposition, and wound remodeling. In addition, Gln-Leu and Asp-Leu pairs, which are very common in the peptides, might act as radical scavengers, and Arg is known to promote collagen accumulation. Additionally, hydrophobic aminoacids, that improve immunity, accounted for more than half of the total aminoacids in the peptides. Interestingly, a similar ameliorated healing quality was obtained by the gastric administration of *P. martensii*’s mantle peptide preparations to mouse skin wound models [140].

**Figure 20:**
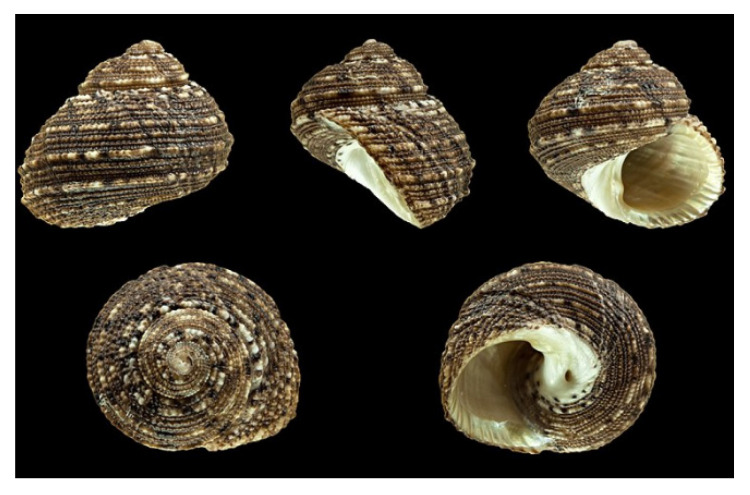
Images of the shell of *Euchelus asper*. © H. Zell (CC BY-SA 3.0); https://commons.wikimedia.org.

*Euchelus asper* (Gmelin, 1791; Gastropoda, Seguenziida: Chilodontaidae; Figure 20) is an eatable and shell-oriented benthic mollusc that is typical of tropical areas and distributed in the Indo-West Pacific region, populating the west coast and south-east coast of India and the rocky beaches near the low tide points [141]. It is characterized by a thick and conoidal dull shell with brown, rosy, and black dots. Ether-soluble fractions of this organism were able to induce immunostimulated phagocytosis in vitro and immunosuppressive activity in vivo [142,143], whereas methanol extracts have been shown to exert an anti-osteoporotic effect in vivo, as indicated by the prominent anti-osteoclastogenic activity and reduction in bone loss induced by the lack of estrogen in a ovariectomised mice model [144]. Agrawal et al. [145] demonstrated that methanol preparations from E. asper displayed strong anti-angiogenic potential in CAM assays due to their inhibitory role on mesodermal blood vessel migration and compartmentalization resulted, at least in part, from a decreased ECM remodelling by MMPs. As a confirmation to this hypothesis, the methanol extracts were also found to down-regulate the activity of MMP-2 and -9 in A549 lung cancer cells and, since these enzymes are involved in the process of cell migration and invasion, A549 cell motility in scratch wound healing assays was consistently reduced.

**Figure 21:**
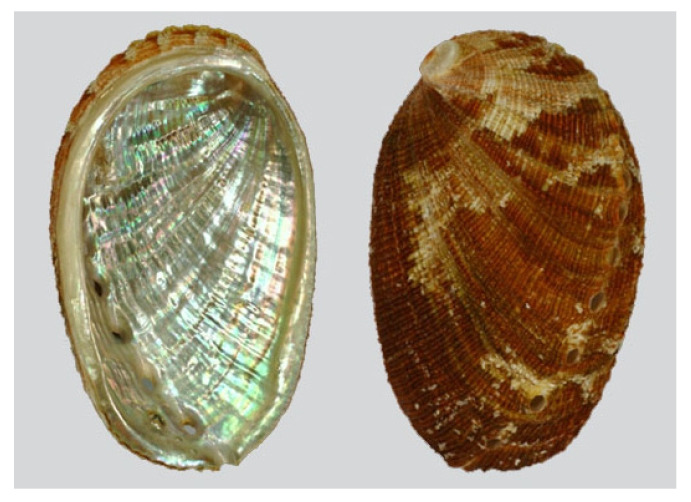
Images of the shell of *Haliotis diversicolor* squamata. © Natural History Museum Rotterdam (CC BY-NC-SA 4.0); http://www.marinespecies.org/.

*Haliotis diversicolor* (Reeve, 1846; Gastropoda, Lepetellida: Haliotidae; Figure 21) is a sea snail that can be found in temperate north-western Pacific Ocean off Japan, Taiwan, Vietnam, western Australia, Indonesian waters, and off New Caledonia [146]. Its coloration is very variable, normally reddish-brown, scarlet, and green in the external part, while the inner surface is silvery with light green and red reflections. It is one of the most important commercial species of molluscs, especially in the southern areas of China. Like most mollusks, H. diversicolor is widely used as a bioindicator for the study of the effects of environmental pollutants or other types of environmental stressors on marine organisms at the cellular and/or molecular level [147,148,149]. The shell of this species, also called “shijueming”, has been considered to be a traditional Chinese medicine used in the past for eye diseases and skin lesions. Xu et al. reported an effective action of this traditional medicine towards the survival rate of human lens epithelial cells damaged by condition of oxidative stress [150], demonstrating its antioxidant and anti-inflammatory properties. Based on those, more recently Chen et al. [151] tested the effect of *H. diversicolor*’s shell powder on inflammation and healing rates of burn injured rats. Their study revealed that the preparation induced a noticeable decrease in neutrophil infiltration, probably due to the down-regulation of nitric oxid synthase, and a significant improvement in the wound healing process with the formation of a well-arranged collagen matrix stimulated by the increase of TGF-β1 content during the tissue remodeling phase.

**Figure 22:**
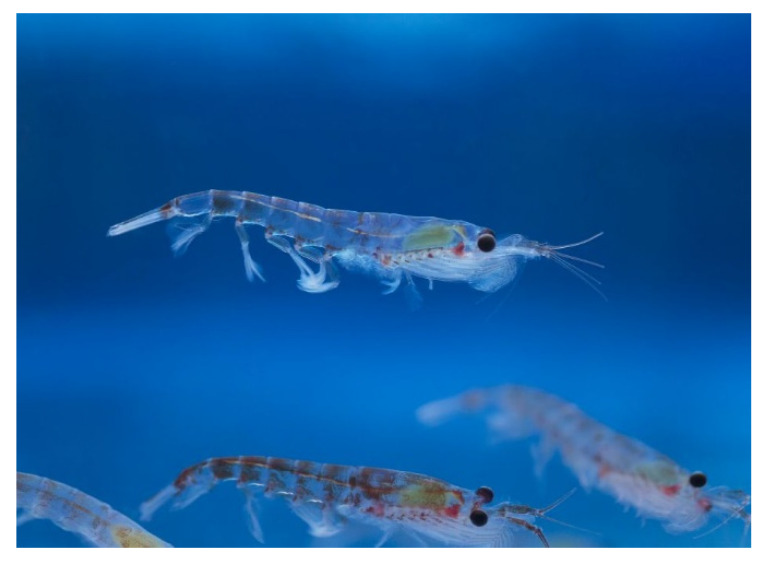
Specimens of *Euphausia superba*. © Brett Wilks; http://www.antarctica.gov.au/.

*Euphausia superba* (Dana, 1850; Malacostraca, Euphausiacea: Euphausiidae; Figure 22) is a pelagic krill that is distributed in the circumpolar belt between the Antarctic continent and the Polar Front [152]. This crustacean is considered to be an economic and ecological keystone [153] for its active involvement in carbon sequestration, iron and nitrogen recycling, ammonium absorption, and conversion of phytoplankton into animal proteins by connecting the different trophic levels [154,155]. The study of the cold adaptation mechanisms of *E. superba* at a transcriptome level has given interesting results that are useful for the design of new and improved recombinant enzyme variants that are endowed with adaptability to cold temperatures [156]. Much interest has been focused on the high nutritional content of this organism, due to the presence of proteins showing a higher biological value than milk or other animal counterparts, a remarkable amount of long-chain, in particular, ω-3, PUFAs, which makes it the second most popular source after fish oil, and all nine essential amino acids thus meeting the FAO/WHO requirements for human health [157,158]. For this reason, krill oil, which is rich in eicosapentaenoic and docosahexaenoic acids, vitamin A and E, and the carotenoid astaxanthin [159], is widely used as a food supplement also in light of its ability to reduce the risks of different pathologies, such as coronary heart diseases, diabetes, hypertension, osteoporosis, inflammation, autoimmune diseases, metabolic syndrome, tumors, and neurological diseases [160,161,162,163]. Costanzo et al. [164] have tested the ability of krill oil to ameliorate the state of intestinal inflammation in an in vitro model while using HT-29 and Caco-2 cell lines. In particular, when confluent cells, mimicking the intact barrier of the gut epithelium, were submitted to scratch wound healing assay in the presence of a mixture of the pro-inflammatory tumour necrosis factor-α and interferon-γ cytokines, the co-exposure to krill oil determined an increase of the rate of cell layer reconstitution. This result, complemented by the evidence of a better recovery of intercellular adhesion systems with a reduction of inflammation-related actin stress fibers, a decrease of cell death, and an impairment of adhesiveness and invasiveness of luminal bacteria on cells, strongly supports the value of krill oil as a prospective drug against human chronic intestinal inflammation. The beneficial effect of krill oil was supposed to be mostly based upon its richness in this lipid component due to the absence of ω-3 PUFAs in corn oil, used as a negative control for the study.

## 5. Echinodermata

**Figure 23:**
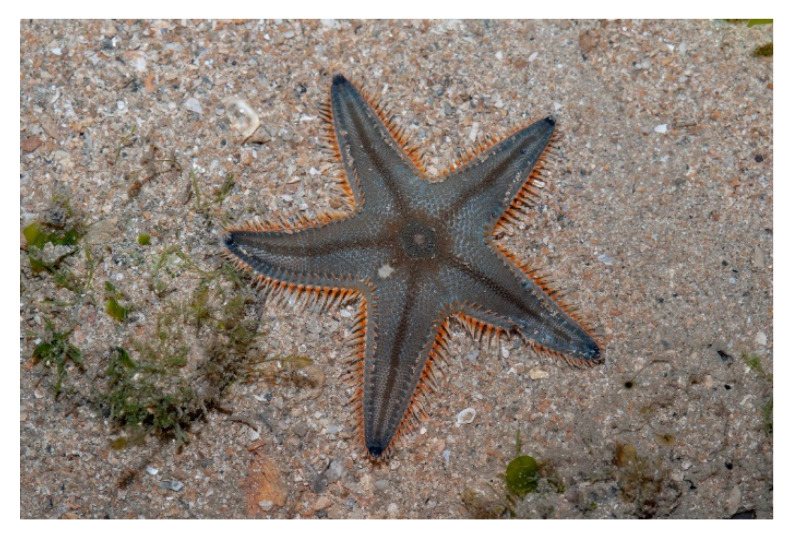
An individual of *Astropecten indicus*. © Ria Tan (CC BY-NC-ND 2.0); https://www.flickr.com/.

The phylum Echinodermata, gathering invertebrates that display a radial symmetry, incorporates about 6000 living marine species around the globe. In recent years, echinoderms have attracted much attention as producers of biomolecules with promising anti-microbial, anti-protozoal, anti-fungal, and anticancer properties, making them potential candidates for drug development and future therapeutic applications [165,166,167,168]. *Astropecten indicus* (Döderlein, 1888; Asteroidea, Paxillosida: Astropectinidae; Figure 23), also called comb star or sand star, is a starfish that is distributed in the tropical environments of the Indo-Pacific area, and in particular of the United Arab Emirates, Qatar, Thailand, Gulf of Mannar, and Palk Bay region. It is a benthic echinoderm that is typically found in the sediments of the intertidal and subtidal regions, where it feeds using infaunal molluscs, and represents a prototypic marine organism to study the effects of environmental pollution [169,170]. Recently, Baveja et al. [171] isolated the AiP1 metalloproteinase from the coelomic fluid of *A. indicus* and demonstrated its fibrinolytic potential that is likely mediated by the von Willebrand factor (vWf)-like domain located in its central β sheet. Moreover, the ability of AiP1 to modulate the motile attitude of HaCaT keratinocytes and HEK293 embryonic kidney epithelial cells was checked through scratch wound healing assays demonstrating the additional AiP1-promoting effect on cell migration since vWf is known to sustain cell locomotory behavior. This result was confirmed by the evidence of the up-regulation of N-cadherin, indicative of EMT, in HEK293 cells. The wound healing activity exerted by AiP1, coupled with the increased cell proliferation reported in the same paper, prompt a more detailed study of the still unidentified mechanism of action of this component of *A. indicus*’s coelomic fluid to devise a novel treatment agent for tissue repair.

**Figure 24:**
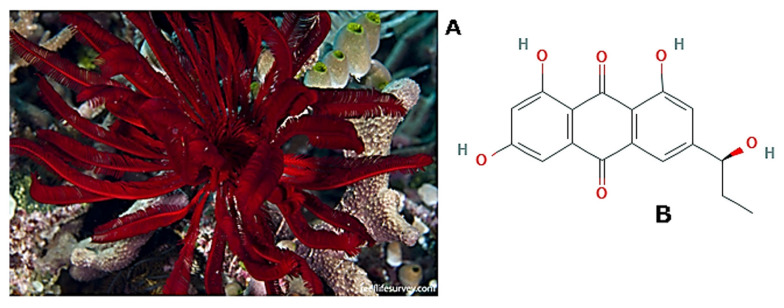
(**A**) An individual of *Himerometra magnipinna*. © Andrew Green; https://reeflifesurvey.com/. (**B**) 2D structure of rhodoptilometrin; https://pubchem.ncbi.nlm.nih.gov/compound/Rhodoptilometrin#section=2D-Structure.

*Himerometra magnipinna* (AH Clark, 1908; Crinoidea, Comatulida: Himerometridae; Figure 24A), also known as *Himerometra robustipinna* (Carpenter, 1881) or red feather star, is a sessile comatulid that lives on hard corals and hard substrates in the Indian and Pacific Oceans, being abundant in the coastal areas of Vietnam and in Nha Trang Bay in the South China Sea [172]. Usually, it has reddish maroon arms and feeds with phytoplankton and zooplankton on detritus. Given that crinoids can regrow arms, internal organs, and entire viscera, the species of this order, including *H. magnipinna*, were the object of considerable studies on the cellular basis of the regeneration process with particular focus on the mechanisms of transdifferentiation [173,174,175,176,177]. A recent paper by Tseng et al. [178] focused on the effect of the *H. magnipinna*-derived anthraquinone (+)-rhodoptilometrin (1,3,8-trihydroxy-6-[(1*S*)-1-hydroxy propyl]anthracene-9,10- dione; Figure 24B) on the migratory behavior of gingival and oral mucosa fibroblasts through scratch wound healing and transwell migration assays. In particular, they found an increased number of migrating cells and improved wound repair rate in the presence of the compound, albeit only limited to gingival fibroblasts. This was coupled to the up-regulation of FAK, fibronectin, and type I collagen, selected as migration-associated ECM-related markers. In the same paper, increased cell viability and glycolysis/oxidative phosphorylation activity, with the latter known to sustain cell mobility functions, were found in rhodoptilometrin-treated human gingival cells, thereby strongly suggesting the potential of the molecule as a beneficial additive in formulations for dental health care and gingival recession treatment.

**Figure 25:**
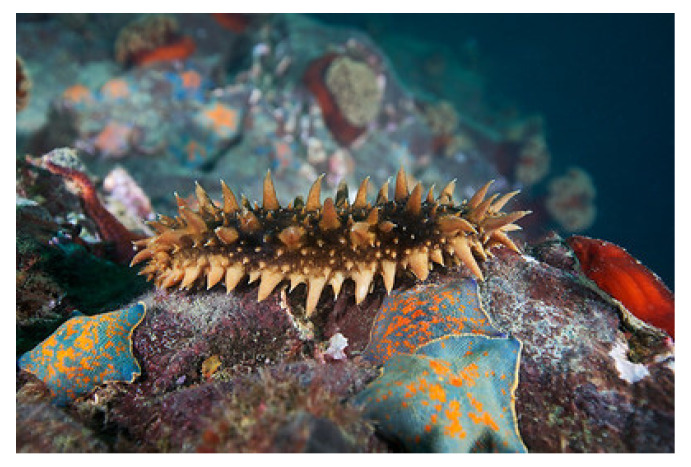
A specimen of *Stichopus japonicus*. © Alexander Semenov; https://www.flickr.com.

*Stichopus japonicus* (Selenka, 1867; Holothuroidea, Synallactida: Stichopodidae; Figure 25), also known as *Apostichopus japonicus* (Selenka, 1867) or Japanese sea cucumber, is a benthic species distributed in the Northwestern Pacific area. It may show three different colors, either red, green, or black, and it populates the shallow coasts from the intertidal zone to depths greater than 100 m. This echinoderm is one of the most important maricultured species in Korea, China, Japan, and several Southeast Asian countries, due to its high market value for human feeding [179]. It is widely recognized that most bioactive properties of sea cucumbers derive from components extracted from their body wall. For example, in the case of *S. japonicus* total extracts exhibited anti-oxidant characteristics, and isolated molecules, such as unsaturated fatty acids and a fucosylated chondroitin sulfate glycosaminoglycan, were proven to exert α-glucosidase inhibitory, immunostimulatory, anti-coagulant, and hypolipidemic activities [180,181,182,183,184]. Collagen, which is the major component of the body wall of *S. japonicus* [185], has been studied for its potential as an additive and functional food ingredient that is capable of maintaining food quality thanks to its gelling properties [186,187]. Park et al. [188] examined the effect of *S. japonicus*’s collagen on keratinocyte biology in vitro. When submitted to scratch wound healing assay, in the presence of this collagen preparation, the HaCaT cell migration rate was higher than that obtained in both bovine gelatin-coated and non-coated plates, being similar to that shown when the cells were seeded onto rat type I collagen substrates. This was accompanied by an up-regulation of fibronectin, as shown by RT-PCR, whose synthesis and extracellular deposition is a recognized fundamental event in the first steps of wound repair. In addition, *S. japonicus*’s collagen increased keratinocyte proliferation positively acting on cell entry in the mitotic phase. Thus, this sea cucumber appears as a valid alternative source with respect to other fish and mammalian species for the extraction of bulky amounts of collagen for biomedical applications, also in light of the documented ability of this collagen to improve skin barrier functions and inhibit melanogenesis, and to play anti-aging, anti-allergic, and anti-wrinkle roles [189,190,191].

**Figure 26:**
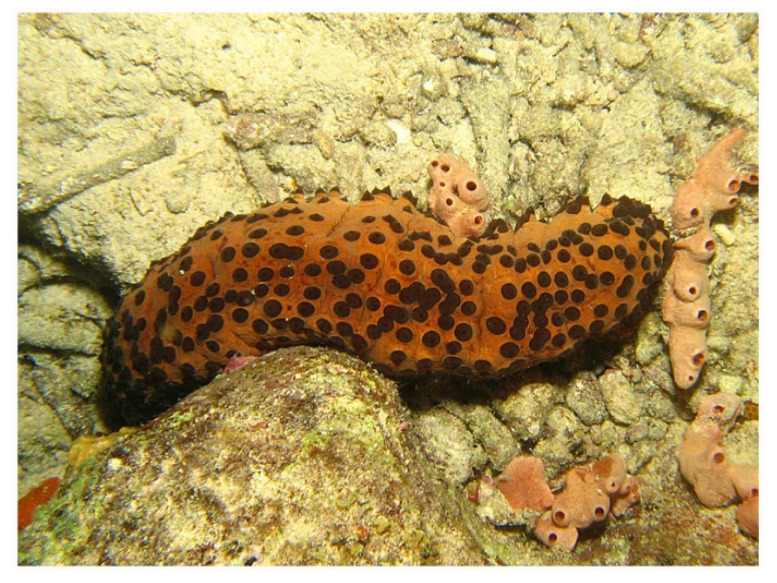
A specimen of *Isostichopus badionotus*. © Dan Hershman (CC BY 2.0); https://commons.wikimedia.org/.

*Isostichopus badionotus* (Selenka, 1867; Holothuroidea, Synallactida: Stichopodidae; Figure 26) is a resource of great economic importance in the south-east of the Gulf of Mexico, Central America and the Caribbean zone. In fact, being rich in fatty acids important for human health [192], it is used as a functional food capable of sustaining growth, improving the lipid profile, and reducing the levels of cholesterol, lipoproteins, and triglycerides in both serum and the liver where it was proven to down-regulate the expression of genes that are associated with dyslipidemia [193]. The most important molecules for biomedical purposes obtained from this sea cucumber, also responsible of the mentioned effects, are fucosylate chondroitin sulphate and fucoidan [194] whose additional properties as anti-coagulant, anti-thrombotic, and anti-malarial compounds have been acknowledged [195,196,197]. Li et al. [198] reported the ability of small molecule oligopeptides that were obtained from I. badionotus by enzymatic hydrolysis to induce the healing of wounds in a diabetic rat model in vivo after back skin section. In particular, in the peptide-treated individuals the wound status displayed a better histological appearance with a higher tensile strength, and the microscopic observations showed increased angiogenesis, collagen deposition, and epithelial reconstitution confirmed by the higher expression of vascular endothelial growth factor (VEGF) in the tissue and more elevated levels of NO and stromal cell-derived factor 1α, both being involved in the secretion of angiogenic factors, in animal sera. Moreover, the peptide treatment favoured the enhancement of antioxidant levels and the reduction of the pro-inflammatory interleukin-6 and -8, tumor necrosis factor-α, chemokine (C-C motif) ligand 2 and C-reactive protein, counteracted by the up-regulation of the anti-inflammatory interleukin-10. This indicates that the natural peptide mixture from I. badionotus sea cucumber might have promising applications for the treatment of diabetic wound healing, which still represents a significant clinical problem.

**Figure 27:**
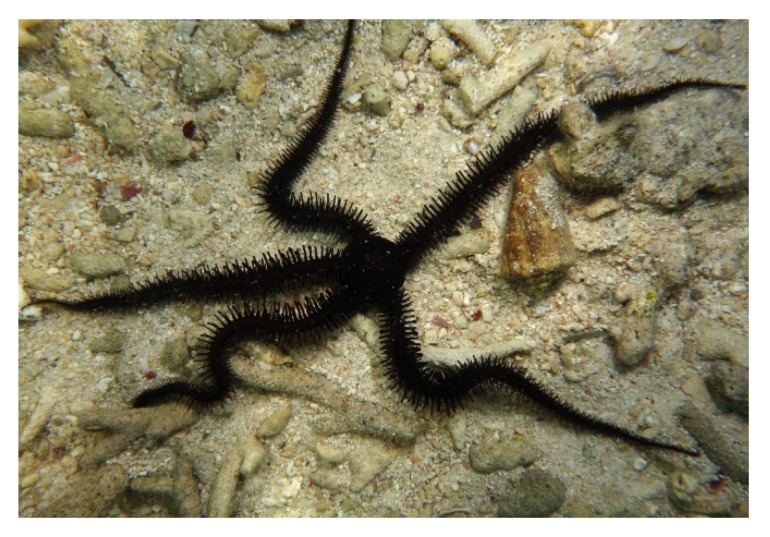
An individual of *Ophiocoma erinaceus*. © Patrick Randall (CC BY-NC-SA 2.0); https://www.flickr.com/.

*Ophiocoma erinaceus* (Müller & Troschel, 1842; Ophiuroidea, Ophiacanthida: Ophiocomidae; Figure 27), also known as brittle star, is typical of tropical- and reef-associated environments and is distributed in the whole Indo-Pacific area (except for Pakistan and western India) being dominant in the Persian Gulf and in the coast of South Africa [172,199]. In the search of novel anti-angiogenic compounds, *O. erinaceus*’s alcoholic extracts, which were endowed with anti-oxidant properties, were administered to A2780cp ovarian tumor cells in culture and an in vivo CAM model, demonstrating a significant inhibition of cell growth and a down-regulation of formation of new blood vessels, coupled to the decrease of VEGF, TGF-β, and FGF-β mRNA expression in endothelial cells [200,201]. Among the components of the extract, an isolated polysaccharide and a saponin fraction were subjected to further studies. In fact, the first compound was found able to exert the previously-mentioned effects, and also decrease the viability of HUVEC cells, their expression of paxillin and MMP-9 mRNAs, whose products are involved in metastatization, and migration in scratch wound healing assays [202]. More recently, the brittle star polysaccharide was also proven to accelerate the wound healing in a male Wistar rat model in vivo [203]. On the other hand, the brittle star saponins appeared to be responsible for the radical scavenging function of the extract via increase of the SOD level in exposed cervical cancer HeLa cells. The survival rate of this cell line was also decreased due to the triggering of the intrinsic apoptotic pathway, differently from that found with non-tumoral fibroblasts. Moreover, saponins impaired cell locomotion in wound healing assay in vitro, parallelly reducing the adhesion of cells to matrigel substrates and inducing the down-regulation of MMP-2 and MMP-9 [204,205]. Of note, brittle star saponins also exhibited an anti-inflammatory ability both in in vitro assays and on macrophages that were differentiated from THP-1/M monocytes, as evidenced by the observed suppression of pro-inflammatory cytokines gene expression and production [206]. Thus, the active principles present in extracts from *O. erinaceus* appear to be promising candidates against human cervical carcinoma and in the prevention of angiogenesis-related disorders.

**Figure 28:**
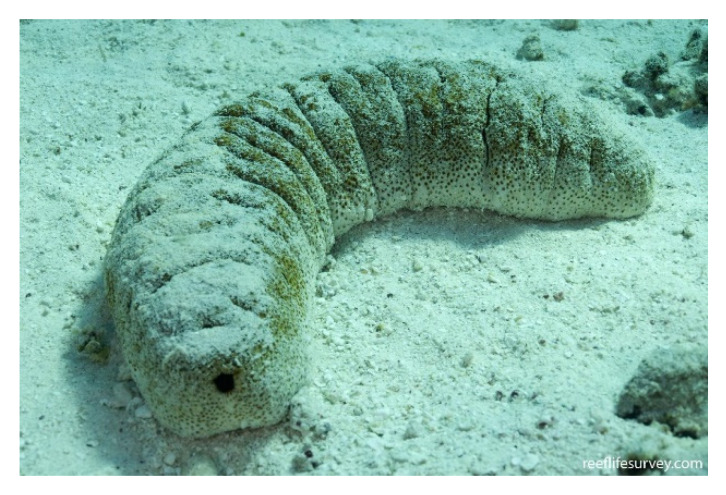
A specimen of *Stichopus herrmanni*. © Andrew Green; https://reeflifesurvey.com/.

*Stichopus herrmanni* (Semper, 1868; Olothuroidea, Synallactida: Stichopodidae; Figure 28), also known as curryfish herrmanni, is a benthic species distributed in the Indo-West Pacific region, being dominant in tropical shallow waters, which represents an important fishery commodity. This orgamism, previously known as *Stichopus variegatus* in Indonesia and Malaysia, has found use for food consumption and preparation of traditional medicines in Asian countries, and for environmental monitoring, e.g., the quantification of marine pollution by polycyclic aromatic hydrocarbons [207,208]. The tegument of this invertebrate is known to contain high amounts of collagen and glycosaminoglycans that have been the object of researches. Biocomposites of hydroxyapatite and *S. herrmannii*’s collagen have been proven to increase the number of neo-formed osteoblasts during bone healing and regeneration, with no toxic side effects [209]. Zohdi et al. [210] prepared a *S. hermannii*’s extract-containing hydrogel dressing (“Gamat Hydrogel”), which, when applied to burn skin lesions in rats, improved wound contraction and closure, aided by a reduced inflammatory reaction, with signs of more efficient re-epithelization at the histological observation. The glycosaminoglycan component in S. herrmannii extracts was shown to play a role as a positive modulator of inflammation also in the healing process of oral mucous traumatic ulcers in rats [211]. More recently, Arundina et al. [212] demonstrated the stimulating effects of the extracts, which are also rich sources of soluble mediators, on the growth behavior and osteogenic differentiation of mesenchymal stem cells, thereby confirming that preparations from S. herrmannii may have beneficial effects on the acceleration of the wound healing and tissue regeneration processes.

## 6. Tunicata

Tunicates (subphylum Chordata) are marine organisms that are covered by a “tunic”, i.e., a tough body shield which mainly incorporates a highly crystalline cellulose nanofiber called tunicin [213,214], and proteins that contain 3,4,5-trihydroxyphenylalanine (TOPA) and pyrogallol amino acid [215,216]. Tunicates are known to be among the richest sources of biologically-active compounds together with sponges and bryozoans [217,218] and possess a very efficient mechanism of healing allowing for the repair of tunic injuries in sea water, a wet and salty environment similar to human body fluid [213]. In particular, at the pH of marine water (8.2) pyrogallol is oxidized and provides the key adhesion functionality forming covalent cross-links with tunicin, which has 120 gigapascals of stiffness and contributes to the mechanical support of the tunic, other TOPAs, or proteins [214]. These pyrogallol-mediated bonds favor the seal of the torn tunic and the control of bleeding. In light of such observations, a limited number of studies have been addressed to the identification of tunicate compounds active on cell migration in vitro and in vivo and tissue repair.

**Figure 29:**
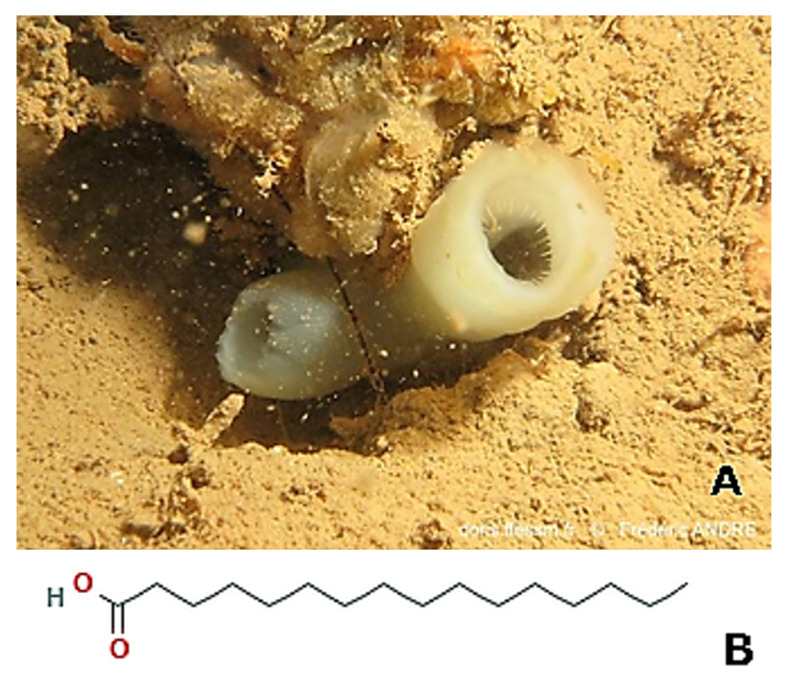
(**A**) An image of *Phallusia arabica*. © Frédéric André; https://doris.ffessm.fr/. (**B**) 2D structure of n-hexadecanoic acid; https://pubchem.ncbi.nlm.nih.gov/compound/Palmitic-acid#section=2D-Structure.

*Phallusia arabica* (Savigny, 1816; Ascidiacea, Phlebobranchia: Ascidiidae; Figure 29A) is a dark grey large solitary ascidian that populates the Red Sea, and the North Atlantic and South Pacific oceans. Ethanolic extracts of *P. arabica* exhibited a positive activity on the skin repair rate in excision rat models in vivo and a significant enhancement of the contraction in incision wounds with a faster re-epithelization and collagen deposition period [219]. It is supposed that the beneficial effect of the ascidian extract lies in the abundant presence of flavonoids, whose anti-inflammatory, anti-fungal, anti-oxidant, and wound healing properties are acknowledged, and the anti-oxidant n-hexadecanoic (palmitic) acid [220] (Figure 29B).

**Figure 30:**
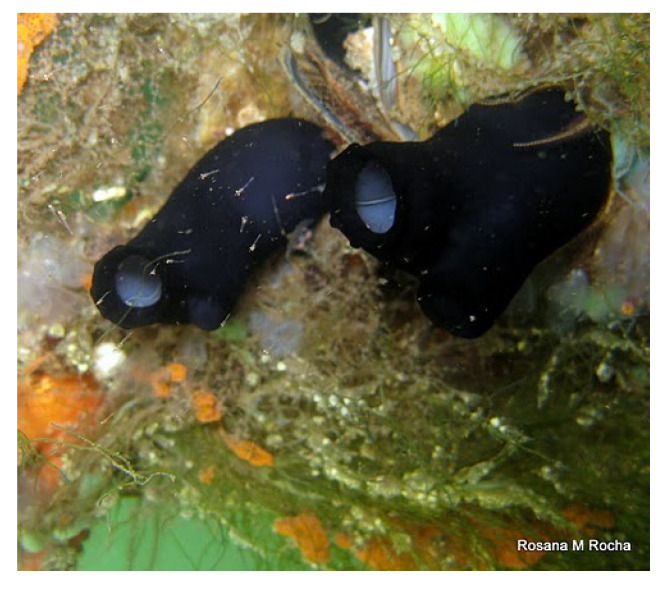
An image of *Phallusia nigra*. © Rosana M. Rocha; http://invasions.si.edu/.

*Phallusia nigra* (Savigny, 1816; Ascidiacea, Phlebobranchia: Ascidiidae; Figure 30) is a velvet black large solitary ascidian, very common in the warm waters of the western Atlantic, ranging from Bermuda to Brazil, as well as in other seas like the Red Sea, Gulf of Aden, and Gulf of Guinea. It occurs as the major component of fouling communities on the hull of ships, piers, pilings and harbour installations. Also the methanolic extract of P. nigra exhibited a beneficial effect towards wound healing in in vivo rat models, similar to those that were obtained with P. arabica extracts, and also in this case the positive influence on tissue repair speed and quality was ascribed to the flavonoid and n-hexadecanoic acid components of the preparation [221,222].

**Figure 31:**
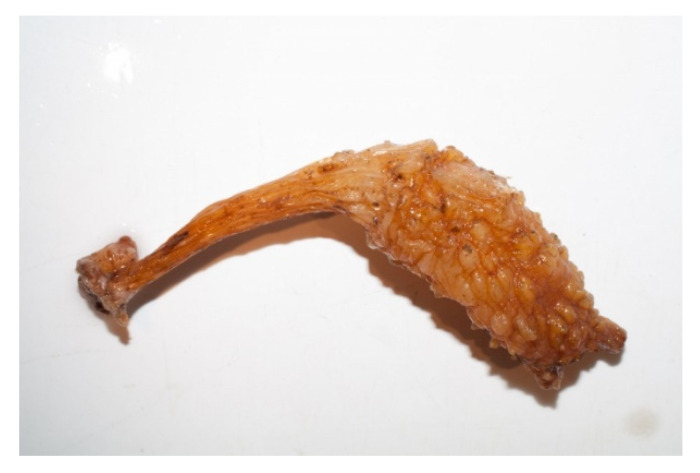
An image of *Styela clava.* © Claude Nozères (CC BY-NC-SA 4.0); http://www.marinespecies.org/.

*Styela clava* (Herdman, 1881; Ascidiacea, Stolidobranchia: Styelidae; Figure 31), also known as stalked sea squirt, is a solitary ascidian displaying a brown or yellow rough and wrinkled surface which is native of the Northwestern Pacific area and it has become a conspicuous member of fouling communities in Northwestern European waters. The cellulose films manufactured from *S. clava*’s tunic (SCT) have been the object of research [223]. These films exhibited good biocompatibility and degradability [224] and, when regeneration and angiogenesis in the surgical wounds of Sprague–Dawley rats were examined after the application of three different preparations of SCT-cellulose hydrogels, the one regenerated from freeze-dried membranes appeared to be a better dressing support when compared to regenerated plain and alginate-supplemented hydrogels [225]. This was due to the highest strength and lowest elongation level being exhibited by this preparation and the consequent amelioration of granulation tissue formation, angiogenesis, and skin reconstitution in the absence of toxic effects. At the molecular level, the reconstituted freeze-dried membranes appeared to up-regulate the levels of VEGF, collagen and TGF-β1, with activation of p-Smad 2/3 signalization, in the damaged tissue. More recently, Song et al. [226] studied the effect of selenium-loaded STC-cellulose films on cutaneous wounds of diabetic rats, showing, also in this experimental system, the acceleration of the healing process with the complete recovery of re-epithelization and skin repair following the activation of the downstream signaling pathways of VEGF, angiopoietin-2/1, and insulin, and the restoration of anti-oxidative status. These data demonstrate a potential use of the *S. clava*-derived material as a beneficial supplementary compound in formulations for topical application to cutaneous wounds.

## 7. Conclusions

An assortment of key events in development, health, and disease depends on collective cell motility which represents a common theme joining morphogenesis, tissue regeneration, and tumor biology [1]. It is widely acknowledged that several signal transduction and molecular mechanisms underlying and critical for cell migration and re-epithelization in wound repair are highly similar to those that are involved in epithelial tumor ingrowth and metastasis. As for medical treatments, it is known that, in some cases, anticancer agents may show interference with wound healing and, in turn, drugs used for non-healing ulcers of diabetic patients may promote carcinogenesis. Thus, there are many molecular and physiologic points in common between wound closure and tumor progression [227]. On the other hand, over the years, the literature showed the rapid rising of the prevalence, severity, and complexity of both cancer and wound healing pathologies, with the consequent remarkable increase in healthcare costs. It is imperative for modern society the procurement of alternative sources of bioactive compounds to replace overexploited resources and, on the other hand, improve treatments and the quality of life of patients via novel and more efficacious drugs. Marine invertebrates are a still poorly exploited valuable resource of natural products that may significantly improve the process of skin regeneration and restrain tumor cell migration, as documented by the in vitro and in vivo studies presented in this review and summarized in a synoptic way in Table 1. Only a very limited number of compounds and methods of extraction have been patented and/or introduced for treatment schemes to date, i.e., krill oil preparations [228], cellulose films from *S. clava* (patent KR20150117748A South Korea), *S. herrmanni*’s collagen for biocomposites [229], and *S. japonicus*’s fucosylated chondroitin sulfate (patent CN102367285A China). In conclusion, there is still an enormous potential offered by marine invertebrates towards a variety of applications that may significantly improve human health and the well-being of future generations. Therefore, the advancement of knowledge in this direction will result in new approaches that involve the identification of the most promising candidates for the utilization as new extracts/molecules, also in combination with existing drugs, which are targeted for biomedical translation.

## Figures and Tables

**Table 1:** Anti-cancer and wound healing-promoting properties of molecules and crude extracts from marine invertebrates.

Effects	Phylum	Species	Compounds	References
**Anti-cell migration/invasion properties**	**Porifera**	*Jaspis stellifera* *Negombata magnifica* *Pseudoceratina arabica* *Suberea mollis* *Siphonocalina (Callyspongia) siphonella* *Xestospongia* sp.	Stellettin B Latrunculin B and 15-O-methyl-latrunculin B Latrunculin A Bromotyrosine derivative (1’R,5’S,6’S)-2-(3’,5’-dibromo-1’,6’ -dihydroxy-4’-oxocyclohex-2’-enyl) acetonitrile Moloka’iamine Ceratinin A Ceratinin D hydroxymoloka’iamine subereamolline A Sipholenol A and its aliphatic polar and aromatic ester derivatives Sipholenol A-4-O-3′,4′-dichlorobenzoate derivative Ranieramycins (i.e ranieramycin M)	[42] [47] [48] [53] [54] [62,64] [71]
	**Cnidaria**	*Cladiella australis* *Cladiella pachyclados* *Sarcophyton crassocaule* *Sinularia nanolobata* *Clavularia koellikeri*	Dihydroaustrasulfone alcohol (synthetic precursor of Austrasulfone) Pachycladin A Sclerophytin F methyl ether Polyanthelin A Sclerophytin A Cembrenolide diterpene 13-acetoxysarcocrassolide 11-dehydrosinulariolide Sinularin Clavukoellian A and clavukoellian D-related 4-O-Deacetylparalemnolin D	[76,77,78] [79] [81,82] [83,84] [87]
	**Mollusca**	*Euchelus asper*	Methanol extracts	[145]
	**Echinodermata**	*Ophiocoma erinaceus*	Saponins	[204,205]
**Pro wound-healing properties**	**Porifera**	*Chondrosia reniformis*	Fraction enriched in hydroxyproline- containing collagenous peptides	[33]
	**Cnidaria**	*Cyanea capillata* *Rhopilema esculentum*	Tentacle extracts, Granulin Collagen peptides	[96,97] [103]
	**Mollusca**	*Uroteuthis (Doryteuthis) singhalensis* *Sepia kobiensis* *Sepia officinalis* *Mytilus galloprovincialis* *and Rapana venosa* *Pinctada martensii* *Haliotis diversicolor* *Euphausia superba*	Radical scavenging-carotenoid astaxhantin Chitosan and collagen Collagen Lipid extracts; Aminoacid mixture from protein extract Peptides extracted from *P. martensii*’s mantle *H. diversicolor*’s shell powder Krill oil	[109] [115] [116] [132,133] [140] [151] [164]
	**Echinodermata**	*Astropecten indicus* *Himerometra magnipinna* *Stichopus japonicus* *Isostichopus badionotus* *Ophiocoma erinaceus* *Stichopus herrmanni*	AiP1 metalloproteinase Anthraquinone (+)-rhodoptilometrin Collagen Oligopeptides Polysaccharides “Gamat Hydrogel” (*S. hermannii*’s extract-containing hydrogel dressing)	[171] [178] [188] [198] [203] [210]
	**Tunicata**	*Phallusia arabica* *Phallusia nigra* *Styela clava*	Ethanolic extracts Methanolic extract Cellulose films manufactured from *S. clava*’s tunic (SCT) selenium-loaded STC-cellulose films	[219] [221,222] [225,226]
